# Design, characterization, and application of a mixed metal oxide (Al_2_O_3_–La_2_O_3_–V_2_O_5_) nanocomposite in photocatalytic degradation of methylene blue: a multi-technique approach

**DOI:** 10.1039/d6ra04119a

**Published:** 2026-07-22

**Authors:** Abdullah Al Mamun, Swapan Kumer Ray, Md. Omar Sha Rafi, Shabiba Parvin Shandhi, Riyadh Hossen Bhuiyan, Fariha Chowdhury, Tanvir Muslim

**Affiliations:** a Department of Chemistry, University of Dhaka Dhaka-1000 Bangladesh osrafi@du.ac.bd tmuslim@du.ac.bd; b Bangladesh Council of Scientific and Industrial Research (BCSIR) Dhanmondi Dhaka-1205 Bangladesh

## Abstract

This study introduces the low-temperature wet-impregnation preparation of a ternary Al_2_O_3_–La_2_O_3_–V_2_O_5_ nanocomposite, built as an efficient visible-light-driven photocatalyst for enhanced dye degradation. The effective nanoscale fabrication was demonstrated by the mean granule size of 49 nm for the corresponding catalysts as determined by scanning electron microscopy (SEM) combined with energy-dispersive X-ray spectroscopy (EDS). Through the implementation of UV-vis spectroscopy, the optical band gap energies were revealed to be 2.72 eV indicating high photocatalytic activity. By observing distinctive peaks from 550 cm^−1^ to 1100 cm^−1^ Fourier-transform infrared spectroscopy (FTIR) analysis and peaks from Raman spectroscopy verified the existence of functional groups. To establish nanocomposite stability and crystallinity, further structural and compositional confirmation was conducted using X-ray diffraction (XRD). The crystalline framework is made up of rhombohedral α-Al_2_O_3_, according to X-ray diffraction (XRD). No distinct crystalline La_2_O_3_ or V_2_O_5_ phase was seen. The Scherrer, Monshi–Scherrer, and Williamson–Hall models yielded crystallite sizes of 43.2, 49, and 58.2 nm, respectively. Thermogravimetric analysis (TGA) demonstrated exceptional thermal stability up to 800 °C with a total mass loss of only 1.05% of the nanocomposite. From transmission electron microscopy (TEM), the contour of the nanocomposite was near sphere-shaped and the measured size was 91 nm, confirming the formation of the nano-catalyst. Brunauer–Emmett–Teller (BET) analysis indicated a specific surface area of 3.87 m^2^ g^−1^ with a mesoporous architecture (average pore size 89.9 Å). Under natural sunlight irradiation, the optimized nanocomposite achieved 97.86% degradation of methylene blue (MB) (10 ppm) within 120 minutes at pH 10 with a catalyst loading of 40 mg, following pseudo-first-order kinetics (*R*^2^ > 0.88). The point of zero charge (PZC) was determined to be pH 7.5, consistent with the observed pH-dependent degradation behavior. These results highlight the potential of customized nano-catalysts in solar-driven photocatalytic degradation of organic dye pollutants, offering a productive path toward applications using renewable energy. The improved photocatalytic performance comes from the combined effects of the thermally-robust Al_2_O_3_ support, the electron-promoting La_2_O_3_ component, and the redox-active, narrow-bandgap V_2_O_5_. Together they help with better charge separation and capture of visible light. These results show that the Al_2_O_3_–La_2_O_3_–V_2_O_5_ nanocomposite is a thermally stable, solar-responsive photocatalytic material that could be useful for the degradation of organic dye pollutants.

## Abstract 2

This study introduces the low-temperature wet-impregnation preparation of a ternary Al_2_O_3_–La_2_O_3_–V_2_O_5_ nanocomposite, built as an efficient visible-light-driven photocatalyst for enhanced dye degradation.

## Graphical abstract

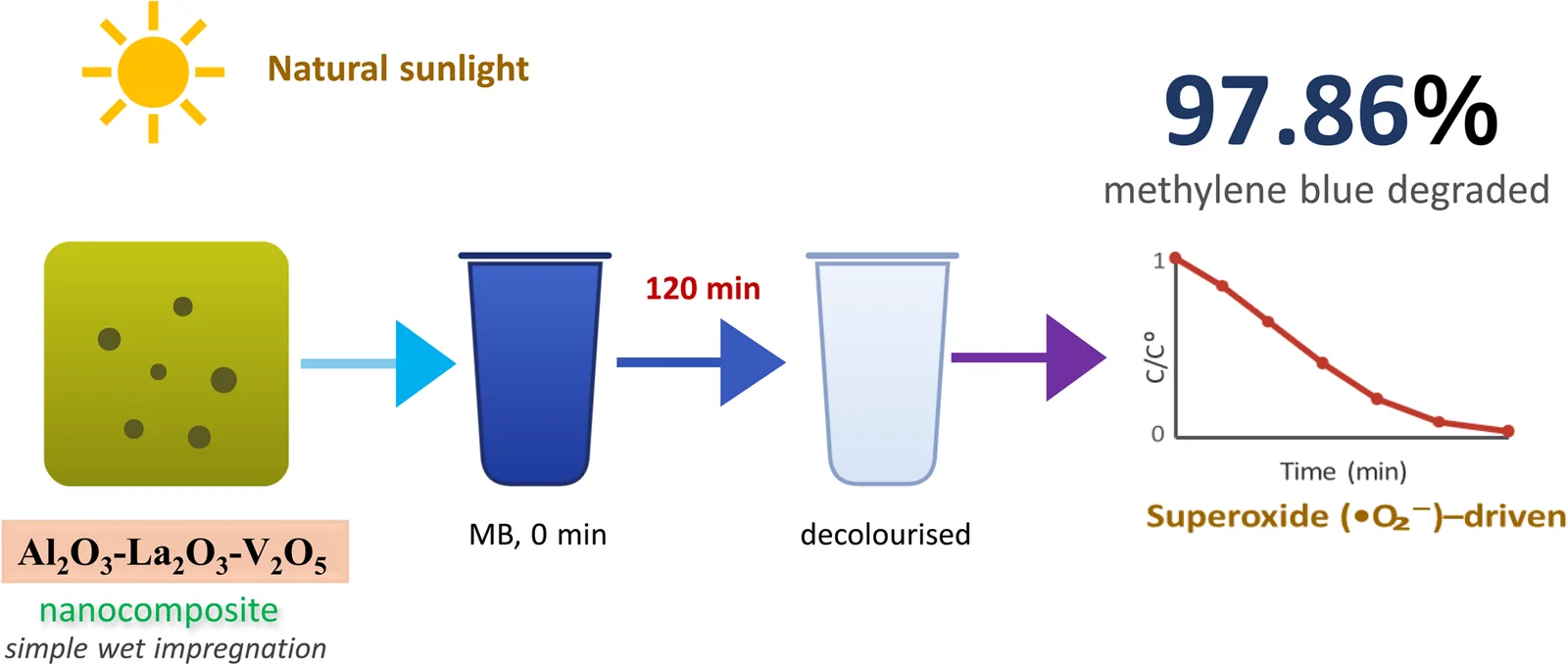

## 1 Introduction

A nanocomposite is a multiphase solid material in which at least one phase has a dimension in the nano-meter range (1–100 nm).^1,2^ Nanocomposite materials provide advantages over microcomposites and monolithics; for example, they have higher strength-to-weight ratio, improved fracture toughness, enhanced thermal and barrier properties, and tunable optical/catalytic behaviors relative to microcomposites and monolithic materials,^3^ but their manufacture presents issues in controlling elemental composition and stoichiometry in the nanocomposite phase.^4^ Nanocomposites can improve the mechanical, thermal, optical, and antibacterial performance of materials in comparison to their bulk counterparts since material properties are highly dimensionality-dependent.^5^ Additionally, nanocomposites are used for storage applications that require high capacity, such as shock protection and durability.^6^ Certain metal–oxide nanocomposites also exhibit antibacterial activity, which is relevant to biomedical and water-disinfection applications.^7^ The widespread use of a range of chemicals, particularly color pollutants from sectors such as leather, textiles, medicines, and cosmetics, has turned water bodies into polluted reservoirs. Textile industry effluents, which contain a variety of organic, inorganic, polymeric, and elemental contaminants, constitute a serious danger to water quality, aquatic biodiversity, soil fertility, and ecosystems in general.^8,9^ In the midst of these issues, nanotechnology has emerged as a viable answer, providing sophisticated catalytic processes that can effectively reduce water pollution.^10^ By encouraging interfacial charge separation and increasing light absorption through cooperative interactions between two semiconductors, binary oxide heterostructures somewhat mitigate these disadvantages.^11^ Since the addition of a third component results in more heterojunction interfaces, better charge transport, increased surface activity, and improved structural stability all of which improve photocatalytic performance ternary oxide nanocomposites have garnered more attention in recent years.^12^ Although various wastewater treatment systems are now in use, such as flocculation, oxidation, and reverse osmosis, many of them are hampered by lengthy procedures, high operational costs, and low efficiency.^13,14^ In contrast, photodegradation, particularly when enabled by photocatalysis, offers a quick, cost-effective, and efficient alternative for water treatment.^15,16^ By increasing the specific surface area and forming a mesoporous network, nano-structuring improves the absorption and degradation of pollutants by increasing the density of accessible active sites.^17^

Combining a semiconductor with a dielectric substance (Al_2_O_3_) led to synergistic effects that increased photocatalytic efficiency.^18^ Photocatalytic destruction of organic contaminants was widely researched.^19,20^ Rare earth metal oxides (REMOs) provide remarkable physical and chemical properties.^21–23^ REMO nanoparticles small size and high surface-to-volume ratio make them suited for a wide range of applications that bulk materials cannot support. Lanthanum oxide (La_2_O_3_), a rare-earth oxide, exhibits strong basicity, oxygen mobility, and the capacity to change the surface acidity–basicity balance, thus improving catalytic activity and resistance to deactivation. Lanthanum oxide (La_2_O_3_) and other rare earth metal oxides (REMOs) have attractive characteristics that make them acceptable as dielectric materials.^24^ Vanadium oxides with different stoichiometries have unique crystal shapes and physical/chemical characteristics. The large range of oxidation states, from V^2+^ to V^5+^, contributes to the diversity observed. Vanadium pentoxide (V_2_O_5_) is the most stable chemical.^25^ Compared to other semiconductors, vanadium oxide has high potential in water.^26^ This treatment is effective due to its light absorption, narrow bandgap, chemical stability, and surface catalytic characteristics. V_2_O_5_ catalyst powder has shown significant photocatalytic efficiency against several organic contaminants.^27–30^ We hypothesized that a single nanocomposite containing Al_2_O_3_, La_2_O_3_, and V_2_O_5_ would offer a platform that combines adsorption and catalytic oxidation, allowing for improved photocatalytic dye degradation. Such synergistic interactions can considerably boost the degradation efficiency of persistent organic contaminants.

Mixed-metal–oxide nanocomposites in general can be prepared by a variety of routes, including combustion,^31^ hydrothermal,^32^ laser ablation,^33^ mechanochemical,^34^ sol–gel,^35^ template method,^36^ microwave-assisted,^37^ Pechini method,^38^ precipitation method,^39^ solvothermal,^40^ pyrolysis,^41^ and ball-milling.^42^ However, these synthetic approaches are highly expensive, possibly hazardous, and necessitate extensive reaction times, dangerous chemical precursors, and specialized experimental equipment. Consequently, these routes have a negative impact on the ecology. This highlights the urgent need to replace or change chemical preparation approaches in order to produce a sustainable, clean, non-toxic, cost-effective, and environmentally friendly process *via* wet impregnation method.

Our study presents the rational design of a ternary Al_2_O_3_–La_2_O_3_–V_2_O_5_ nanocomposite as a visible-light-driven photocatalyst for degradation of organic dye pollutants. The fundamental goal of this research was to create a structurally stable nanocomposite and rigorously determine the relationship between crystallite structure, morphology, optical band gap, and photocatalytic degradation ability. The prepared nanocomposite displayed better degrading efficiency against methylene blue, indicating its promise as a sustainable and solar-responsive material for degradation of organic dye pollutants.

## 2 Materials and methods

### 2.1 Materials

Reagents such as aluminum oxide (CAS: 1344-28-1, Sigma-Aldrich, USA), lanthanum oxide (CAS: 1312-81-8, Sigma-Aldrich, USA), vanadium pentoxide (CAS: 1314-62-1, Sigma-Aldrich, USA), and nitric acid (CAS: 7697-37-2, Sigma-Aldrich, USA), MB (CAS: 61-73-4 (methylene blue, anhydrous), Sigma-Aldrich, USA) were supplied by the Bangladesh Council of Scientific and Industrial Research (BCSIR). All reagents were used without further purification. All the chemicals were used exactly as purchased and were AnalaR grade.

### 2.2 Methods

#### 2.2.1 Preparation of Al_2_O_3_–La_2_O_3_–V_2_O_5_ nanocomposite

Before the preparation of the composites, the metal oxide supports Al_2_O_3_ were calcinated in an electric furnace at 500 °C for 3 hours to eliminate any remaining organic species or adsorbed impurities, enhance structural stability. For the Al_2_O_3_–La_2_O_3_–V_2_O_5_ nanocomposite, where Al_2_O_3_ served as the support material, 10.0 g of Al_2_O_3_ was dispersed in 15 mL of 1.0 M nitric acid. Then, 0.5 g each of La_2_O_3_ and V_2_O_5_ were added to this solution with stirring to ensure proper mixing. The mixture was first heated at 60 °C for 3 hours with continuous stirring at 500 rpm to facilitate uniform mixing and partial dissolution. Following this, the temperature was increased, and the mixture was further heated at 150 °C with stirring at 600 rpm for approximately 18 hours.

**Fig. 1:**
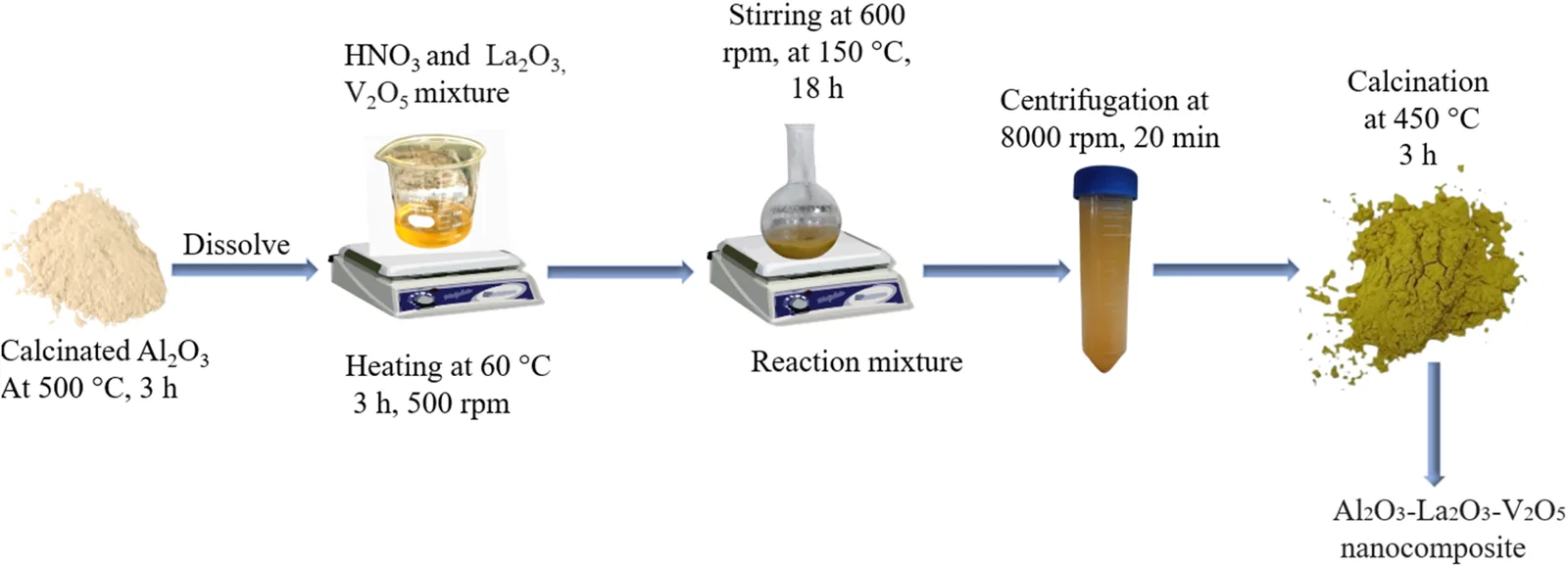
A schematic diagram of the synthetic pathways Al_2_O_3_–La_2_O_3_–V_2_O_5_ nanocomposite.

After heating, the solutions were centrifuged at 8000 rpm for 20 minutes in order to separate the solid composites. The separated solid was collected without washing and calcined directly in static air; the residual nitrate detected by FTIR (Section 3.1) is consistent with this procedure. The collected composites were then calcined in a muffle furnace at 450 °C for 3 hours to complete the synthesis (Fig. 1). These synthesized composites were then stored in airtight containers for further characterization and application studies.

In this study the Al_2_O_3_ : La_2_O_3_ : V_2_O_5_ mass ratio was fixed at 10 : 0.5 : 0.5, with Al_2_O_3_ as the majority support and La_2_O_3_ and V_2_O_5_ as minor functional components; optimisation of the component ratio was beyond the present scope and is a subject for future work.

#### 2.2.2 Fourier transform infrared spectroscopy (FTIR)

The IR spectra of Al_2_O_3_–La_2_O_3_–V_2_O_5_ nanocomposite were recorded using an FTIR spectrophotometer (IRPrestige-21; Shimadzu, Japan). The pellets were manufactured with powdered metal oxide and spectroscopic grade KBr, which is a diluting medium. An FT-IR spectrometer was used to measure transmittance in the 4000–400 cm^−1^ wavenumber range, with a resolution of 4 cm^−1^ and 16 scans per test. All of the infrared bands were interpreted and correlated with the chemical species using the absorption bands recorded elsewhere.

#### 2.2.3 UV-visible spectroscopy

A UV-visible spectrophotometer (Cintra 2020, GBC Scientific Equipment, Australia) was used to characterize the electronic spectra of synthesized Al_2_O_3_–La_2_O_3_–V_2_O_5_ nanocomposite. Solid-state UV-visible spectra were taken at one-hour intervals to ensure reproducibility until no more absorbance changes were seen. Absorbance spectra were recorded in the wavelength range of 250–800 nm.

#### 2.2.4 Thermogravimetric analysis

The precursor was thermogravimetrically analyzed (PerkinElmer, Pyris 1 TGA, Germany) to determine the temperature at which it would convert to Al_2_O_3_–La_2_O_3_–V_2_O_5_ nanocomposite, allowing for the prediction of potential chemical changes that would occur during the calcination process. About 5 mg of the sample was subjected to thermal analysis in a platinum pan with nitrogen environment. Heating temperature was 25 °C to 850 °C at a rate of 10 °C increment.

#### 2.2.5 Scanning electron microscopy and energy dispersive X-ray spectroscopy (SEM/EDS)

The surface appearance, particle size, and constituent elemental compositions of produced Al_2_O_3_–La_2_O_3_–V_2_O_5_ nanocomposite were studied using a scanning electron microscope equipped with an energy dispersive X-ray spectrometer (JEOL JSM-7610F, Japan). Prior to analysis, the samples were taped to carbon and sputter-coated with platinum in an inert argon atmosphere. Measurements were conducted using a charge compensator at a 15 kV applied electron voltage.

#### 2.2.6 X-ray powder diffractometer (XRD)

The structural phase analysis of Al_2_O_3_–La_2_O_3_–V_2_O_5_ nanocomposite were performed using an ARL™ EQUINOX 1000 X-ray diffractometer manufactured by Thermo Fisher Scientific in the United States using a monochromatic copper Kα1 radiation (*λ* = 0.1541 nm) with coplanar asymmetric geometry, operated at 40 kV and 15 mA. The reported range of 2*θ* was 20°–80° with a scanning speed of 6° min^−1^. The size of the particles was estimated using Debye–Scherrer's formula.

#### 2.2.7 Raman spectroscopy

Raman spectroscopy studies were performed with a HORIBA MACRO-RAM Raman Spectrometer (HORIBA Scientific, India). The instrument generates high-resolution spectrum data appropriate for studying molecular vibrations and material composition. A laser excitation source was utilized to probe the material, and the scattered light was collected and evaluated within the specified spectral region. Calibration and baseline correction were done with the integrated LabSpec program. All measurements were carried out under controlled conditions to ensure reproducibility and accuracy.

#### 2.2.8 Transmission electron microscopy (TEM)

Al_2_O_3_–La_2_O_3_–V_2_O_5_ nanocomposite was analyzed by Transmission Electron Microscopy (TEM) (Talos F200X G2, Czech Republic). Samples were placed on a carbon-coated copper grid for 1 minute to form a tiny film. Filter paper was used to remove extra liquid, which was then successively placed in a grid box.

#### 2.2.9 BET analysis

Utilizing nitrogen adsorption–desorption isotherm data collected at −196 °C (77 K) on a constant-volume adsorption apparatus utilizing Micromeritics ASAP 2020 Plus 2.00 version, USA. The specific surface areas (SSA) of the samples were determined using Brunauer–Emmett–Teller (BET) theory. Before BET analysis, the samples were degassed for three hours at 200 °C.

#### 2.2.10 Photocatalytic activity

An organic dye, methylene blue (C_16_H_18_N_3_SCl·3H_2_O, MB) was used to evaluate the photocatalytic degradation capacity of the prepared Al_2_O_3_–La_2_O_3_–V_2_O_5_ nanocomposite. All photocatalytic tests were performed outdoors under natural sunlight in Dhaka, Bangladesh (≈23.7°N, 90.4°E) on 21/06/2026 between 11:00 am and 1:00 pm local time, when the solar irradiance measured with a pyranometer was ≈850 W m^−2^; the ambient temperature was ≈31 °C under clear skies, and all runs were carried out within the same daily time window and comparable weather to limit variation in sunlight intensity. To exclude airborne particulate contamination while permitting irradiation, the Pyrex reaction beaker was loosely covered with a transparent watch glass.

The MB concentration was varied (5, 10, 20 and 30 ppm) at fixed catalyst mass and pH; the catalyst loading was varied (10, 20, 40 and 80 mg) at fixed 10 ppm MB and pH; and the pH was varied from 2 to 12 at fixed 10 ppm MB and 40 mg catalyst. The pH was adjusted with 0.1 M HCl and 0.1 M NaOH and monitored with a calibrated pH meter. The photocatalytic degradation process was examined at different reaction times from 0 to 120 minutes at 20-minute intervals. Adsorption/desorption equilibrium was settled after stirring in the dark for thirty minutes. Throughout the experiment, the liquids were kept in suspension by a continuous stirring motion. To extract the nanoparticles from the suspension, it was additionally centrifuged for 15 minutes at 5000 rpm. The absorbance was measured using a Cintra 2020 UV-visible spectrophotometer, Australia. The reaction process was monitored by measuring the light absorbance of the MB solution with a UV-vis spectrophotometer at a wavelength of 664 nm.

## 3 Results and discussion

### 3.1 FTIR spectra of Al_2_O_3_–La_2_O_3_–V_2_O_5_ nanocomposites

**Fig. 2:**
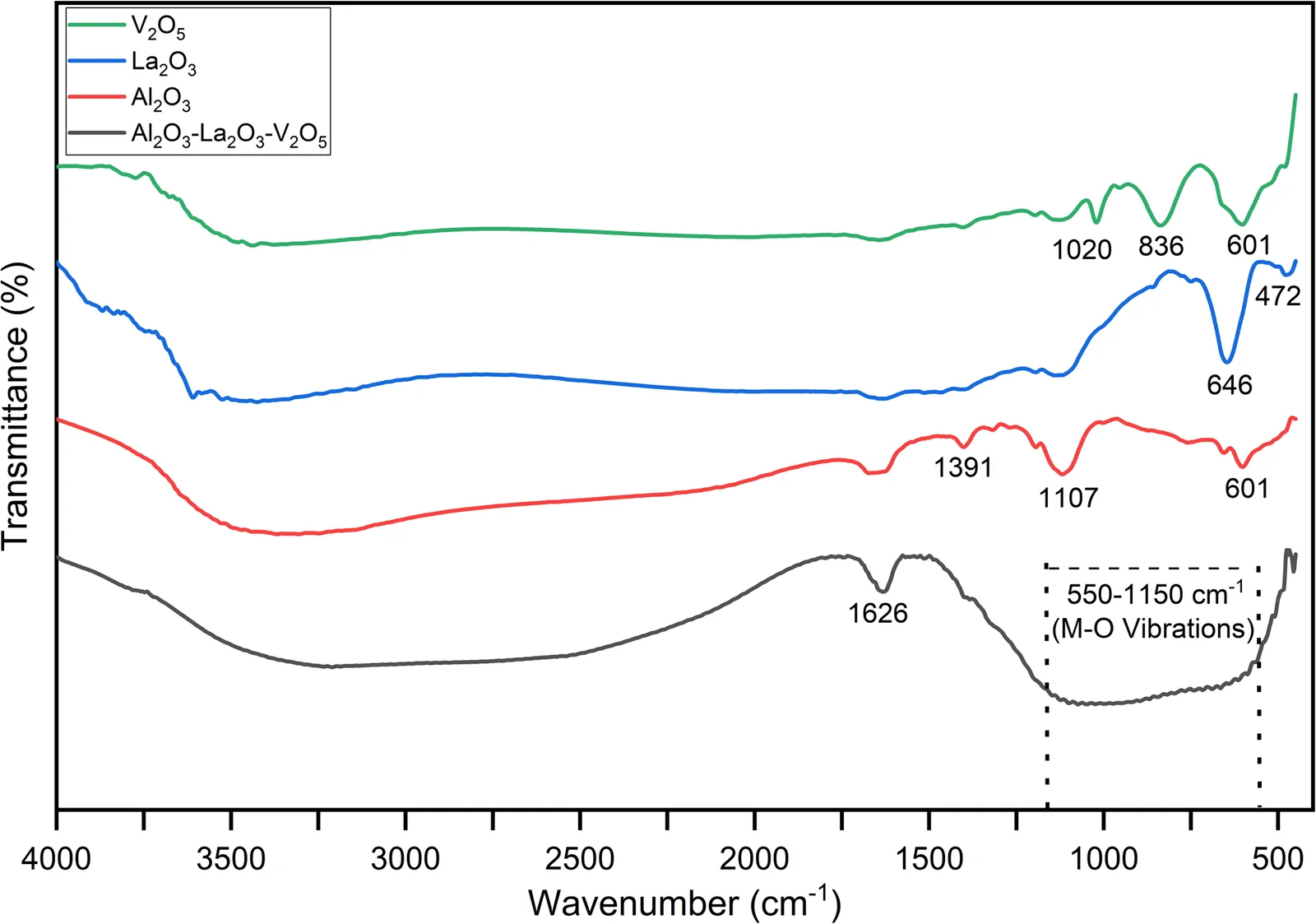
FTIR spectrum of Al_2_O_3_–La_2_O_3_–V_2_O_5_ nanocomposites.

The IR-spectrum in Fig. 2 shows, For V_2_O_5_ spectrum analysis, the vibration at 1020 cm^−1^ is identified as the result of the V=O stretching (vanadyl oxygen). Both vibrations at 836 and 601 cm^−1^ are consistent with V–O–V deformation modes.^43^

For La_2_O_3_ spectrum analysis, the peak observed at 472 cm^−1^ and 646 cm^−1^ belongs to the stretching of La–O and the occurrence of this band at 1120 cm^−1^ implies the presence of surface hydroxyl groups (–OH) or adsorbed substances.^44^ For Al_2_O_3_ spectrum analysis, the primary peaks at 601 cm^−1^ correspond to the Al–O stretching mode in octahedral structure, while the expansive absorbance regions in the spectrum at 1107 cm^−1^ are related to O–H deformation vibrations. The presence of NO_3_^−^ is evident from the spectra at 1391 cm^−1^. Adsorbed water, specifically the H–O–H bending vibration, is the reason behind the peak found at 1650 cm^−1^ in the Al_2_O_3_ FTIR spectra.^45^ The peaks observed at 3480, 3520 and 3456 cm^−1^ belong to the O–H stretching vibration. The overlapping metal–oxygen vibrations of Al–O, La–O, V–O–V, and terminal V=O bonds are responsible for the large absorption band in the 550–1100 cm^−1^ area of the Al_2_O_3_–La_2_O_3_–V_2_O_5_ nanocomposite's FTIR spectrum. This band's broadness suggests that the three oxide components coexist and that their metal–oxygen frameworks may interact. Furthermore, the stretching and bending vibrations of surface hydroxyl groups and adsorbed water molecules are represented by the broad band at 3456 cm^−1^ and the peak at 1626 cm^−1^, respectively.^46–48^

### 3.2 UV-visible spectra of Al_2_O_3_–La_2_O_3_–V_2_O_5_ nanocomposites

**Fig. 3:**
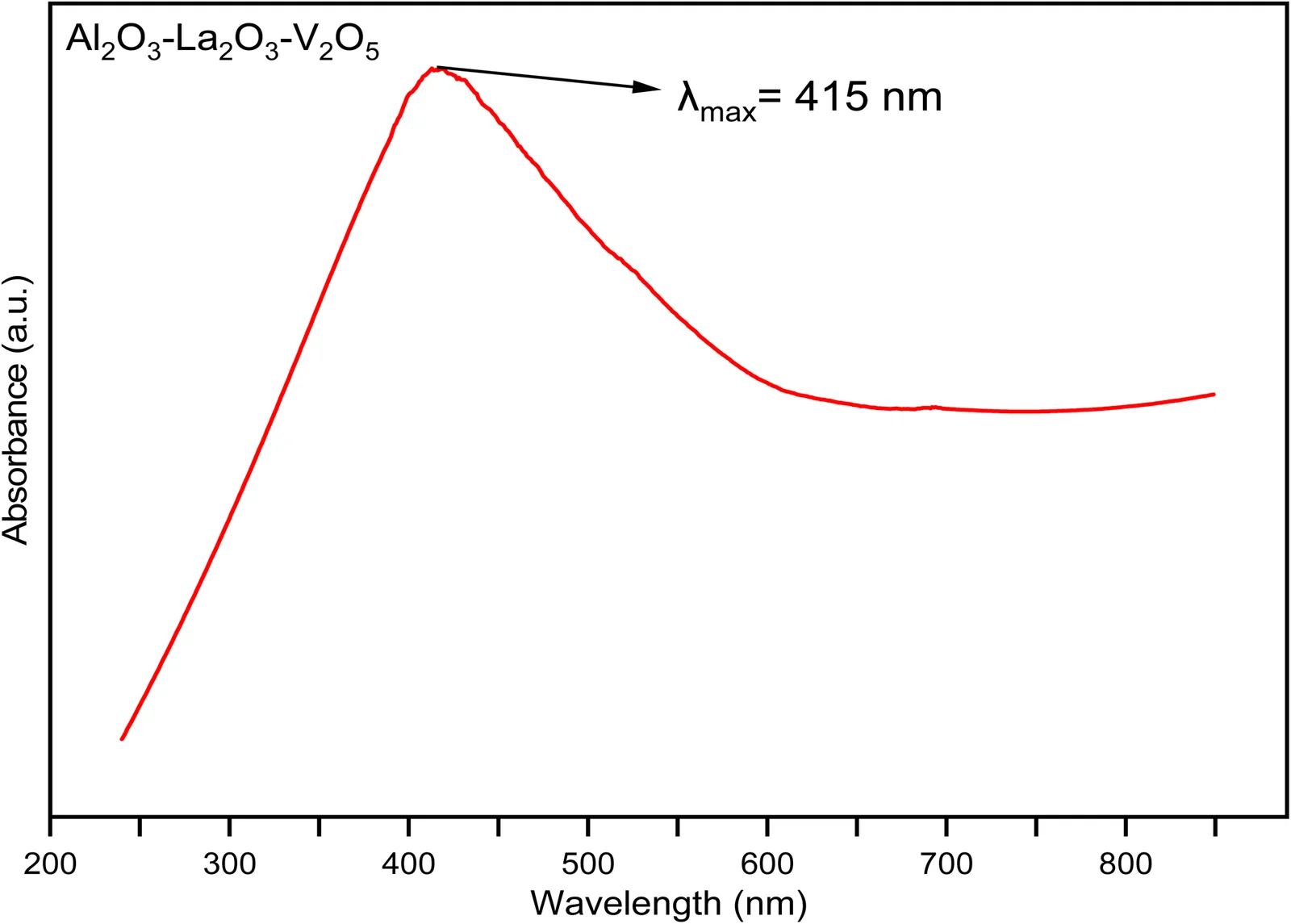
UV-vis spectrum of Al_2_O_3_–La_2_O_3_–V_2_O_5_ nanocomposites.

The UV-vis absorption spectrum was obtained to evaluate the sample optical energy band gap. The Al_2_O_3_–La_2_O_3_–V_2_O_5_ nano composite UV-vis absorption spectra show an absorption band located at 415 nm (Fig. 3). The O^2−^ → V^5+^ ligand-to-metal charge transfer (LMCT) transition of vanadium oxide species is responsible for the absorption band at about 415 nm. The proven electrical transitions of V^5+^ documented in the literature serve as the foundation for this project.^49^ Thus, the band verifies that V_2_O_5_ is present in the composite and that it contributes to the absorption of visible light. UV-vis spectroscopy encompasses visible light up to 800 nm, whereas the UV area is typically defined as below 400 nm. La^3+^ doping alters the local electronic environment, resulting in a minor red-shift of absorption bands. This explains the observed *λ*_max_ at 415 nm.^50^

### 3.3 Tauc plot of Al_2_O_3_–La_2_O_3_–V_2_O_5_ nanocomposites

**Fig. 4:**
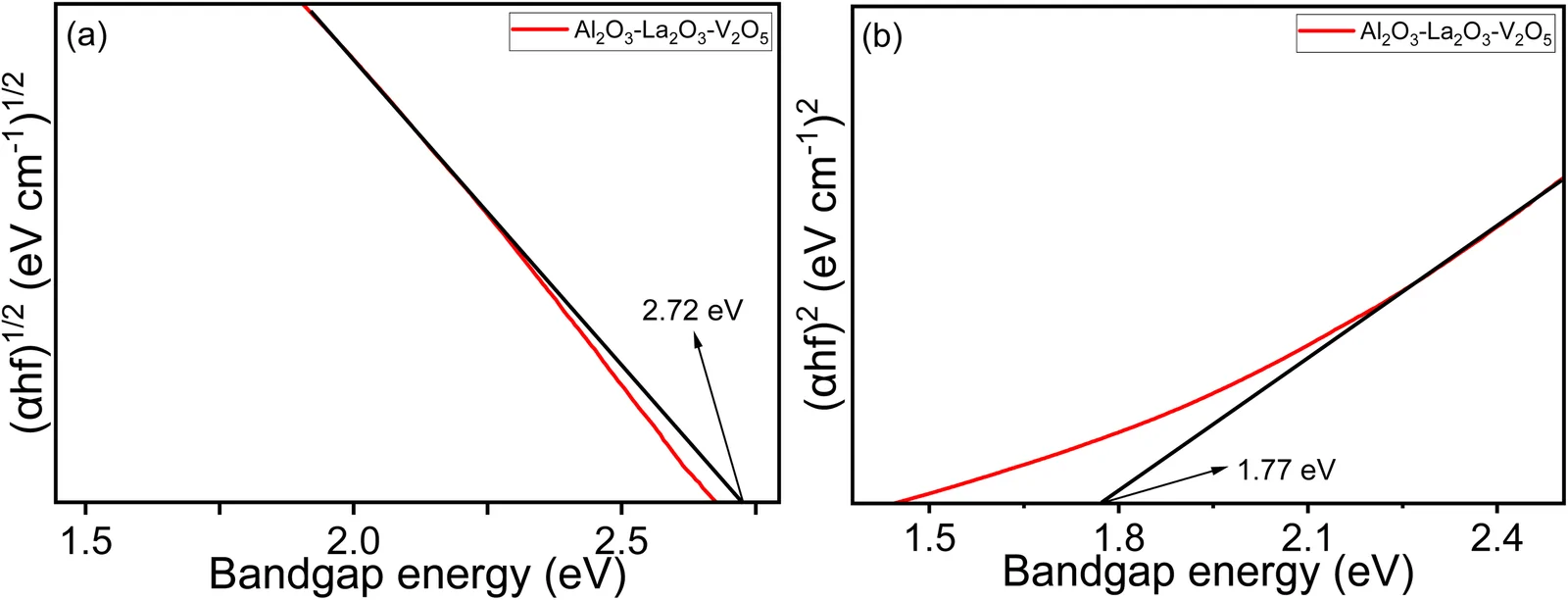
Calculated band gap energy of Al_2_O_3_–La_2_O_3_–V_2_O_5_ nanocomposites (a) direct transition (b) indirect transition.

The images in Fig. 4 reveal a graph comparing (*αhν*)^*n*^ and (*hν*) values of Al_2_O_3_–La_2_O_3_–V_2_O_5_ nanocomposites across all composites.

Tauc equation was used to calculate optical band gaps (*E*_g_) based on photon energy (*hν*).^51^*αhν* = *A*(*hν* − *E*_g_)^*n*^where *A* is the proportionality constant, and *α* is the absorption coefficient, *n* is the exponent factor that determines the nature of the electronic transition generating the absorption. It can take the values both 2 and 1/2, which correspond to direct or indirect transition, respectively.^52^ As illustrated in Fig. 4, the measured data clearly reveal that the band gap value of Al_2_O_3_–La_2_O_3_–V_2_O_5_ nanocomposites is 2.72 eV for direct transition and 1.77 eV for indirect transition. The direct band gap enables effective photoexcitation at higher photon energies, the lower indirect band gap improves visible-light absorption under solar irradiation.^53^ Longer carrier lifetimes are typically produced by direct transitions lower electron–hole recombination rate, which is beneficial for photocatalytic processes. The improved photocatalytic degradation of methylene blue is facilitated by the combined effect, which broadens the optical response and encourages the production of photogenerated charge carriers.^54^

The relatively narrow band gap of the composite suggests the presence of intra-gap defect states, which may enhance visible-light absorption and facilitate charge carrier generation.^55^

### 3.4 Thermogravimetric analysis of Al_2_O_3_–La_2_O_3_–V_2_O_5_ nanocomposites

**Fig. 5:**
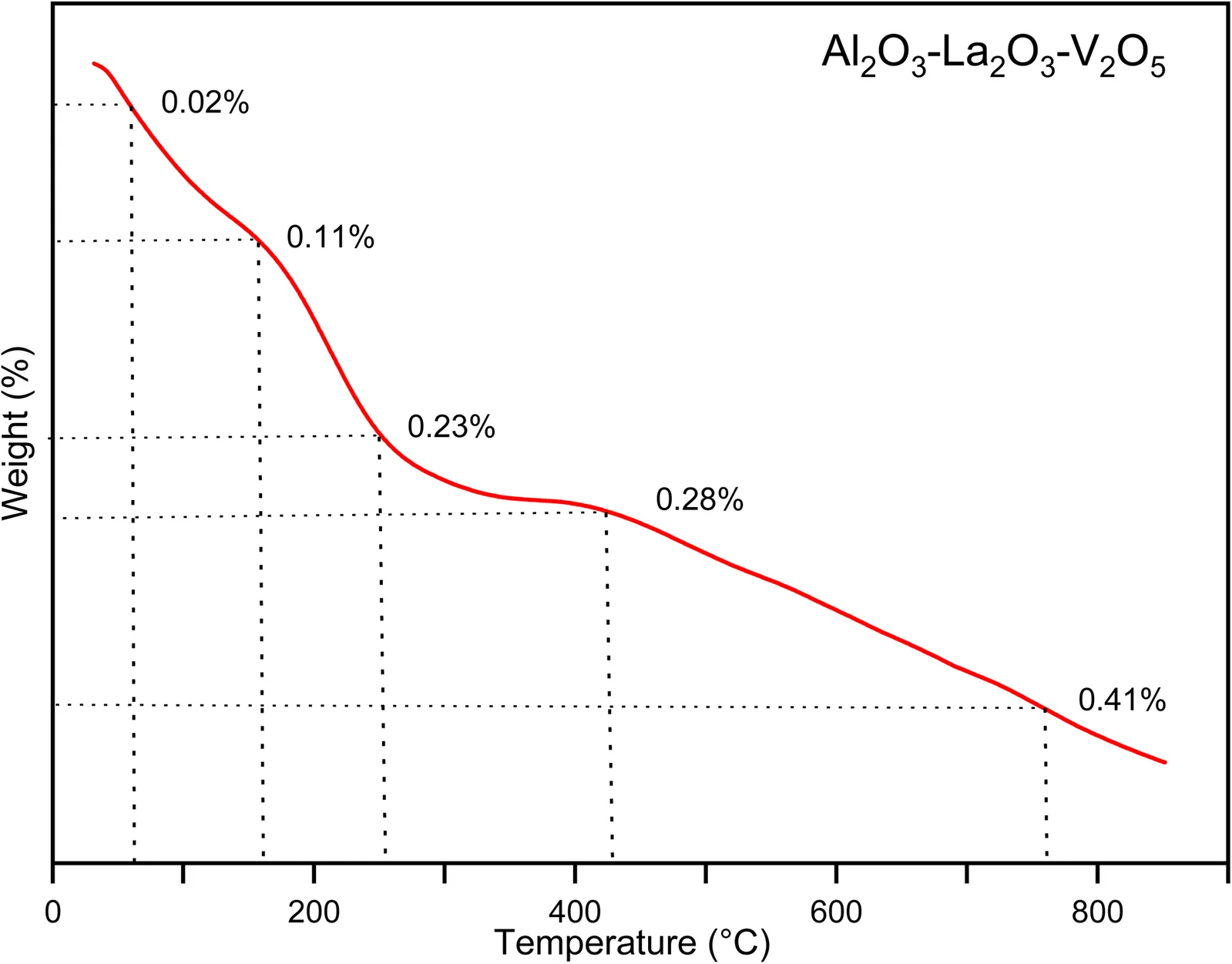
TGA analysis of Al_2_O_3_–La_2_O_3_–V_2_O_5_ nanocomposites.

The thermal stability and weight-loss behavior of the synthesized Al_2_O_3_–La_2_O_3_–V_2_O_5_ nanocomposite were investigated using thermogravimetric analysis (TGA, Fig. 5) under a nitrogen atmosphere from room temperature to 850 °C. The TGA curve showed a total weight loss of about 1.05%, which happened in separate stages corresponding to different temperature ranges.

At 58 °C, the catalyst surface desorbs physically adsorbed moisture, resulting in a small initial weight loss of 0.02%. At 156 °C, a slight loss of 0.11% is likely due to the removal of tightly bound water molecules, such as those trapped in pores or coordinated with metal centers.

A third stage at 252 °C, with a weight loss of 0.23%, may be related to the breakdown of organic contaminants or hydroxyl groups. At 424 °C, an additional 0.28% loss was detected, probably due to partial dehydroxylation of the metal oxide surfaces. The steady loss of 0.41% at 850 °C may be due to structural rearrangements or phase transitions, especially in vanadium or lanthanum species, which have been observed at high temperatures.^56^ The overall minimal weight loss shows that the composite catalyst has exceptional thermal stability, which is advantageous for high-temperature catalytic applications. These observations demonstrate that the synthesized Al_2_O_3_–La_2_O_3_–V_2_O_5_ nanocomposites material maintains structural integrity up to 800 °C with minimal degradation.

### 3.5 Field emission scanning electron microscope (FE-SEM) of Al_2_O_3_–La_2_O_3_–V_2_O_5_ nanocomposites

**Fig. 6:**
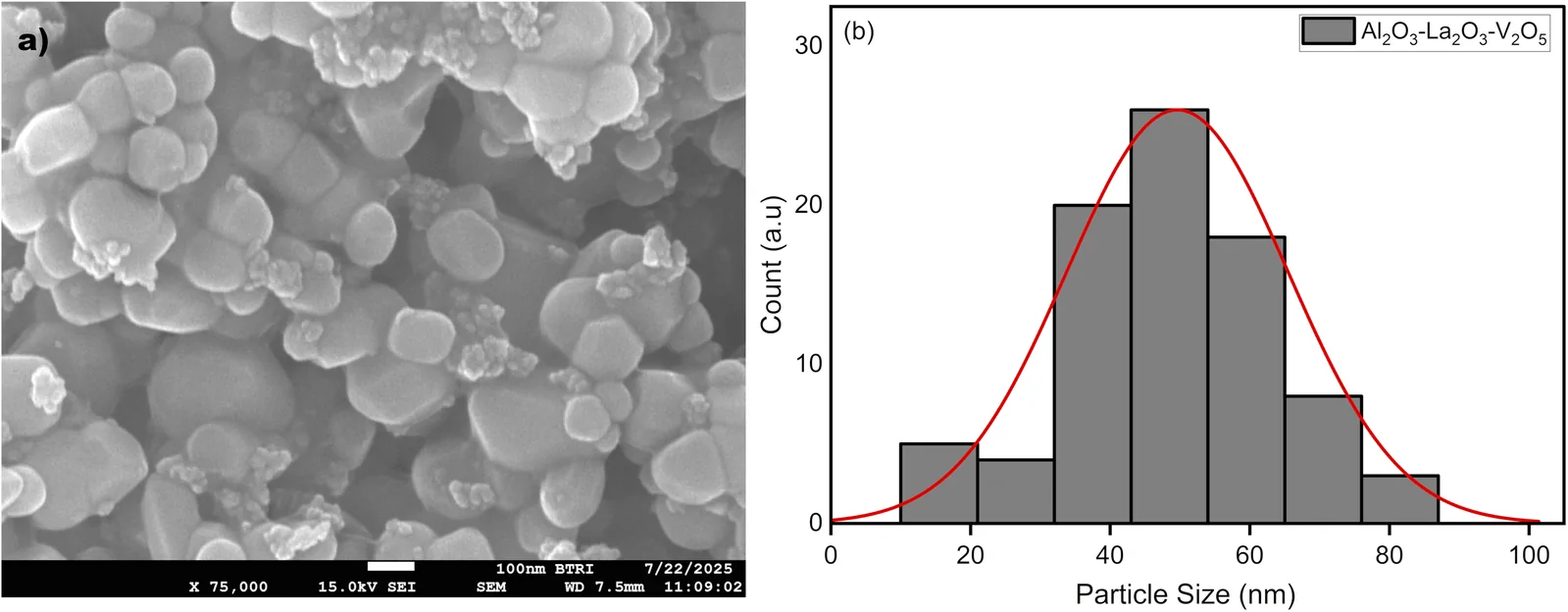
(a) Scanning electron microscopic image and (b) size distribution curve of Al_2_O_3_–La_2_O_3_–V_2_O_5_ nanocomposites.

**Fig. 7:**
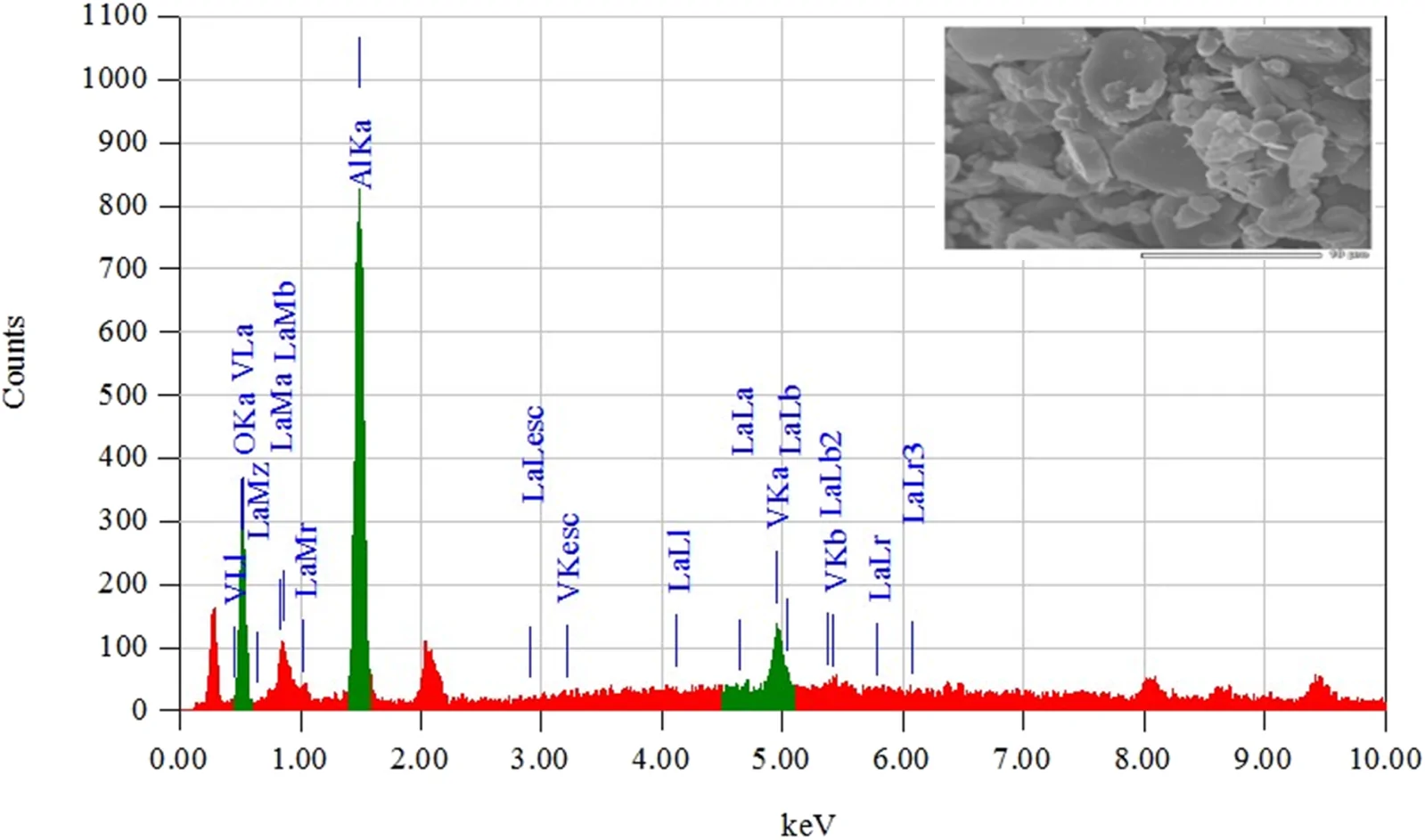
Energy-dispersive X-ray spectroscopy of Al_2_O_3_–La_2_O_3_–V_2_O_5_ nanocomposites.

Fig. 6 shows microscope pictures of the Al_2_O_3_–La_2_O_3_–V_2_O_5_ nanocomposites. The surface morphology of the produced nanocomposite was investigated. The elemental composition and atomic percentages of the Al_2_O_3_–La_2_O_3_–V_2_O_5_ nanocomposites were also determined using the EDS spectrum (Fig. 7).

The nanocomposite outward shape was spherical. The SEM pictures clearly showed randomly scattered, equally formed, spherical-shaped particles that had been aggregated or gathered to create larger particles. The Al_2_O_3_–La_2_O_3_–V_2_O_5_ nanocomposites particles were fairly spherical, with an average size of 49 nm. When the components are combined equally, the nanocomposite size fluctuates. The EDS analysis clearly demonstrated that the produced catalyst is pure Al_2_O_3_–La_2_O_3_–V_2_O_5_ nanocomposites.

#### 3.5.1 Energy dispersive X-ray (EDX) of Al_2_O_3_–La_2_O_3_–V_2_O_5_ nanocomposites

The EDS analysis indicates the probable elemental composition of the manufactured composite catalyst. In the Al_2_O_3_–La_2_O_3_–V_2_O_5_ nanocomposites, the atomic percentages of oxygen were 62.43%, aluminum was 28.17%, vanadium was 8.94%, and lanthanum was 0.45%. The subsequent mass percentages were 43.87%, 33.38%, 20.00%, and 2.75%, respectively (Table 1).

**Table 1:** Element percentage of Al_2_O_3_–La_2_O_3_–V_2_O_5_ nanocomposites

Element	Mass%	Atom%	Sigma
Al	33.38	28.17	0.33
La	2.75	0.45	0.17
V	20.00	8.94	0.16
O	43.87	62.43	0.40

This study did not include spatially resolved elemental mapping; however, the combined SEM–EDS and Raman data show that lanthanum and vanadium species are dispersed throughout the alumina matrix. EDS/STEM elemental mapping is suggested for future research to directly confirm the spatial homogeneity of the constituent elements.

### 3.6 XRD analysis of Al_2_O_3_–La_2_O_3_–V_2_O_5_ nanocomposites

**Fig. 8:**
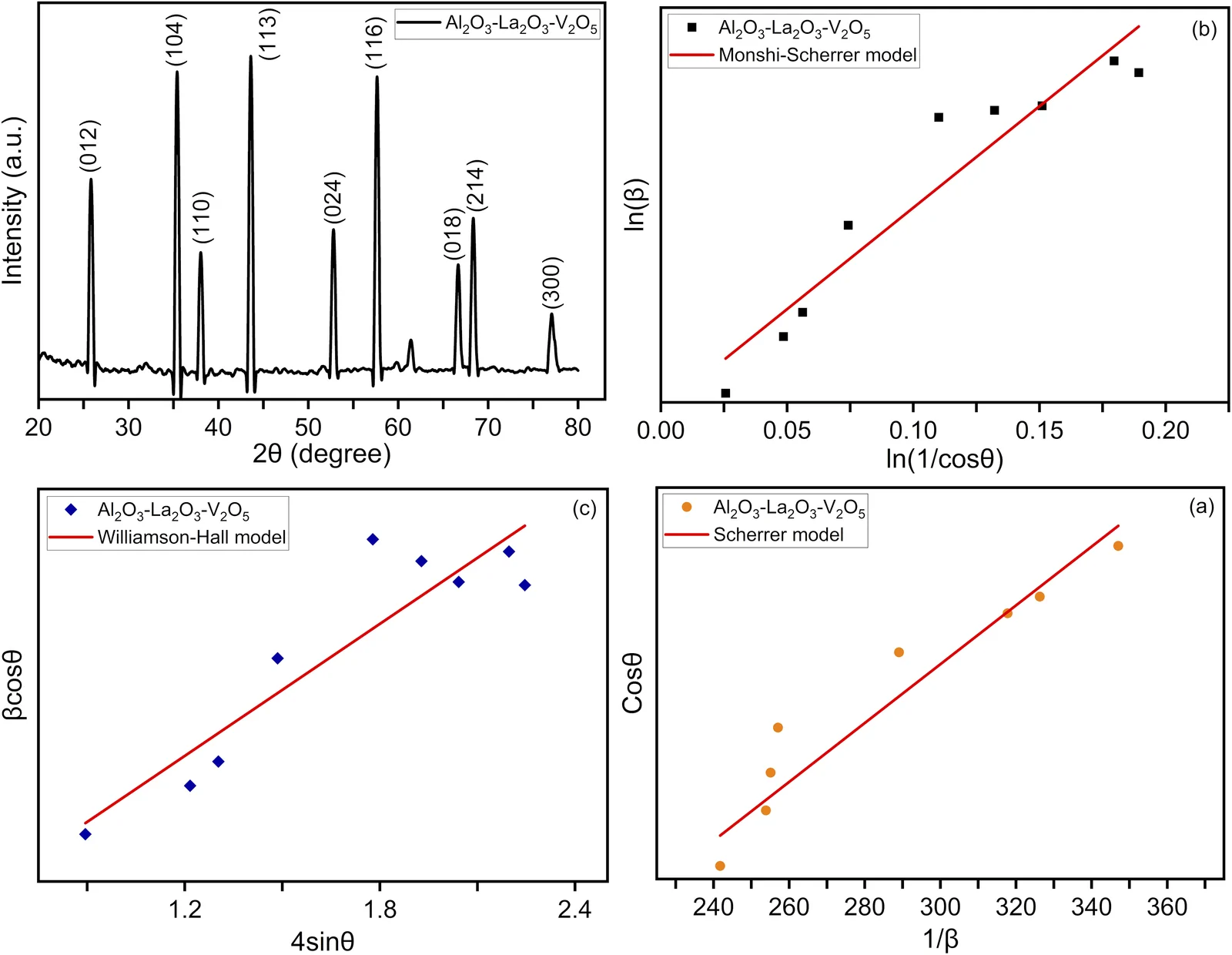
XRD pattern of Al_2_O_3_–La_2_O_3_–V_2_O_5_ nanocomposites (a) Scherrer plot, (b) Monshi–Scherrer plot, and (c) Williamson–Hall plot.

Fig. 8 shows the X-ray diffraction pattern of the produced Al_2_O_3_–La_2_O_3_–V_2_O_5_ nanocomposites. The XRD pattern shows diffraction peaks at 2*θ* of 25.84°, 35.41°, 38.04°, 43.60°, 52.79°, 57.79°, 61.41°, 66.63°, and 68.34°, which correspond to the (012), (104), (110), (113), (024), (116), (018), (214), and (300) crystal planes of rhombohedral Al_2_O_3_ (JCPDS card number: 46-1212).^57,58^ There are no resolved reflections that may be attributed to crystalline La_2_O_3_, V_2_O_5_, or a binary vanadate phase (such as LaVO_4_ or AlVO_4_). The absence of their characteristic reflections, particularly the strong V_2_O_5_(001) line near 2*θ* ≈ 20° and the main La_2_O_3_ reflections at 2*θ* ≈ 26°–30°, indicates that the lanthanum and vanadium species are highly dispersed over the alumina support or present as X-ray-amorphous surface phases below the detection limit of powder XRD. This is expected since alumina makes up the majority of the composite (≈91 wt%). This ternary's phase behavior is in line with Zuev's^59^ reported subsolidus relations, which allow binary compounds like LaVO_4_ and AlVO_4_ to form below 1200 °C. Instead, Raman spectroscopy (Section 3.7), which resolves the terminal V=O mode at 995 cm^−1^ and VO_4_/orthovanadate-type modes significantly more sensitively than XRD, and EDS (8.94 at% V, 0.45 at% La; Section 3.5.1) confirm the presence and loading of the scattered La and V species.

#### 3.6.1 Scherrer plot

The average crystallite size of Al_2_O_3_–La_2_O_3_–V_2_O_5_ nanocomposites was determined using Scherrer's equation as follows.^60,61^1
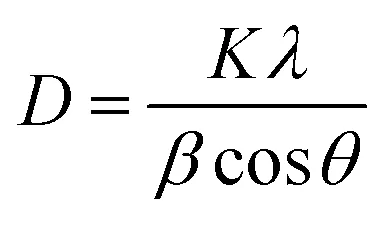
In this equation, *D* represents the crystallite size, *K* is the Scherrer constant (0.9), *λ* is the X-ray wavelength (0.15406 nm), *β* is the peak width of half maximum, and *θ* is the Bragg diffraction angle. The X-ray line broadening method and Scherrer equation were employed to determine grain sizes. Plots were created with 1/*β* on the *X*-axis and cos *θ* on the *Y*-axis, as seen in Fig. 8(a). Crystalline size (*D*) was determined by fitting the data to the slope. The average crystallite sizes for the Al_2_O_3_–La_2_O_3_–V_2_O_5_ nanocomposite was 43.2 nm. Each reflection was fitted with a pseudo-Voigt profile to get peak positions and full-width-at-half-maximum (FWHM) values. The unresolved reflection near 2*θ* = 77° was not included in the size analysis.

When the crystallite size is greater, reduced instrumental broadening allows for more accurate determination of crystallite size. Conversely, when the size of crystallites is relatively small, the signal-to-noise ratio becomes more critical than the instrumental broadening itself.^62^

#### 3.6.2 Monshi–Scherrer plot

Another popular approach for estimating size of crystallites is the Monshi–Scherrer method, which is derived from the Scherrer equation. The Monshi–Scherrer approach fixated on diminishing errors and taking into account all points when estimating crystallite size.^63^ After rearranging the Scherrer equation, the logarithm was taken on both sides. After rearrangement, the equations are represented as eqn (2)–(4). Eqn (4) is generally known as the Monshi–Scherrer equation.2
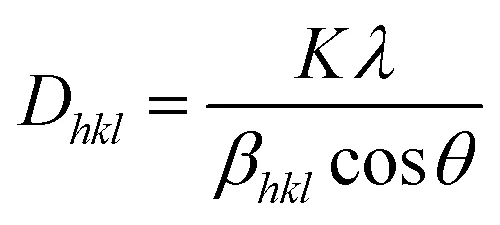
Or,3
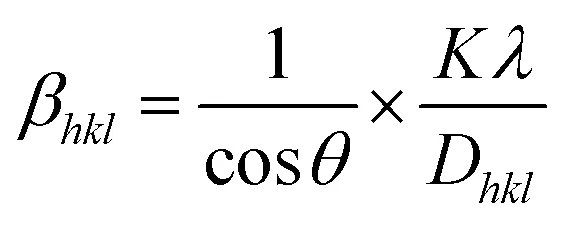
4
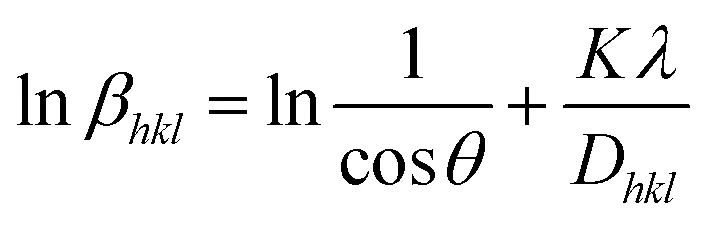


Using this formula, the average crystallite sizes for the Al_2_O_3_–La_2_O_3_–V_2_O_5_ nanocomposites from the intercept was 49 nm. The models gave consistent sizes with good linear fits (*R*^2^ = 0.91 for Monshi–Scherrer and 0.84 for Williamson–Hall).

#### 3.6.3 Williamson–Hall plot

The Williamson–Hall (W–H) plot has been widely used to estimate size of crystallites and strain in crystal-like constituents. This approach expresses the microstructural characteristic when macrostrain and crystal size interact to impact reflection broadening in the XRD pattern.^64^

Williamson and Hall introduced the W–H approach, which uses peak width as a function of 2*θ* to deconvolute size and strain broadening. Fig. 8(c) depicts a graph known as the W–H plot. The plot shows 4 sin *θ* on the *x*-axis and *β* cos *θ* on the *y*-axis (in radians). A linear fit determines the *y*-intercept and slope, which are used to assess particle size and strain.^65^ The Stokes and Wilson method can be represented as the resulting eqn (5), which indicates the strain in the crystals.

5 *β*_strain_ = 4*ε* tan(*θ*)

Rearranging eqn (5), the strain can be represented as eqn (6). In addition, by rearranging the Scherrer equation [eqn (1)], eqn (7) can be derived, which accounts for the contribution of crystallite size to peak broadening.6
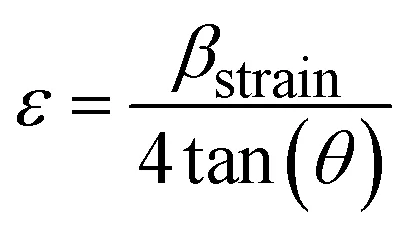
7
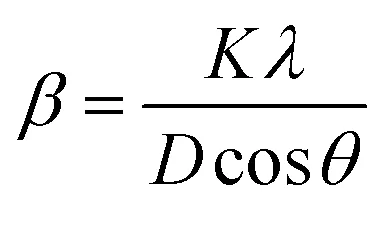
When two constraints, size and strain, influence peak expansion in a sample, the full width at half maximum (FWHM) can be represented as eqn (8). Eqn (6) and (7) are incorporated into eqn (8) to create a new formula eqn (9). The crystallite size induced peak widening varies on cos(*θ*), while the extent of strain-related peak broadening correlates with tan(*θ*).

8 *β*_measured_ = *β*_size_ + *β*_strain_

The equation can be expressed as follows, using the Scherrer and Stokes formula:9
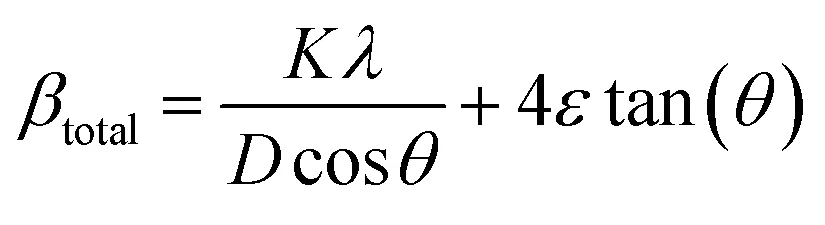


Using this equation, the average crystallite sizes for the Al_2_O_3_–La_2_O_3_–V_2_O_5_ nanocomposites from the intercept was 58.2 nm and the slope gave a small micro-strain of *ε* ≈ 5.0 × 10^−4^.

The three models produced crystallite sizes that were consistent and had acceptable linear fits (*R*^2^ = 0.84 for Williamson–Hall and 0.91 for Monshi–Scherrer). The Williamson–Hall value is slightly higher than the Scherrer estimate because it takes into consideration the minor lattice strain (*ε* = 5.0 × 10^−4^), which, together with crystallite size, contributes to peak broadening in the multiphase composite.

### 3.7 Raman spectroscopy of Al_2_O_3_–La_2_O_3_–V_2_O_5_ nanocomposites

**Fig. 9:**
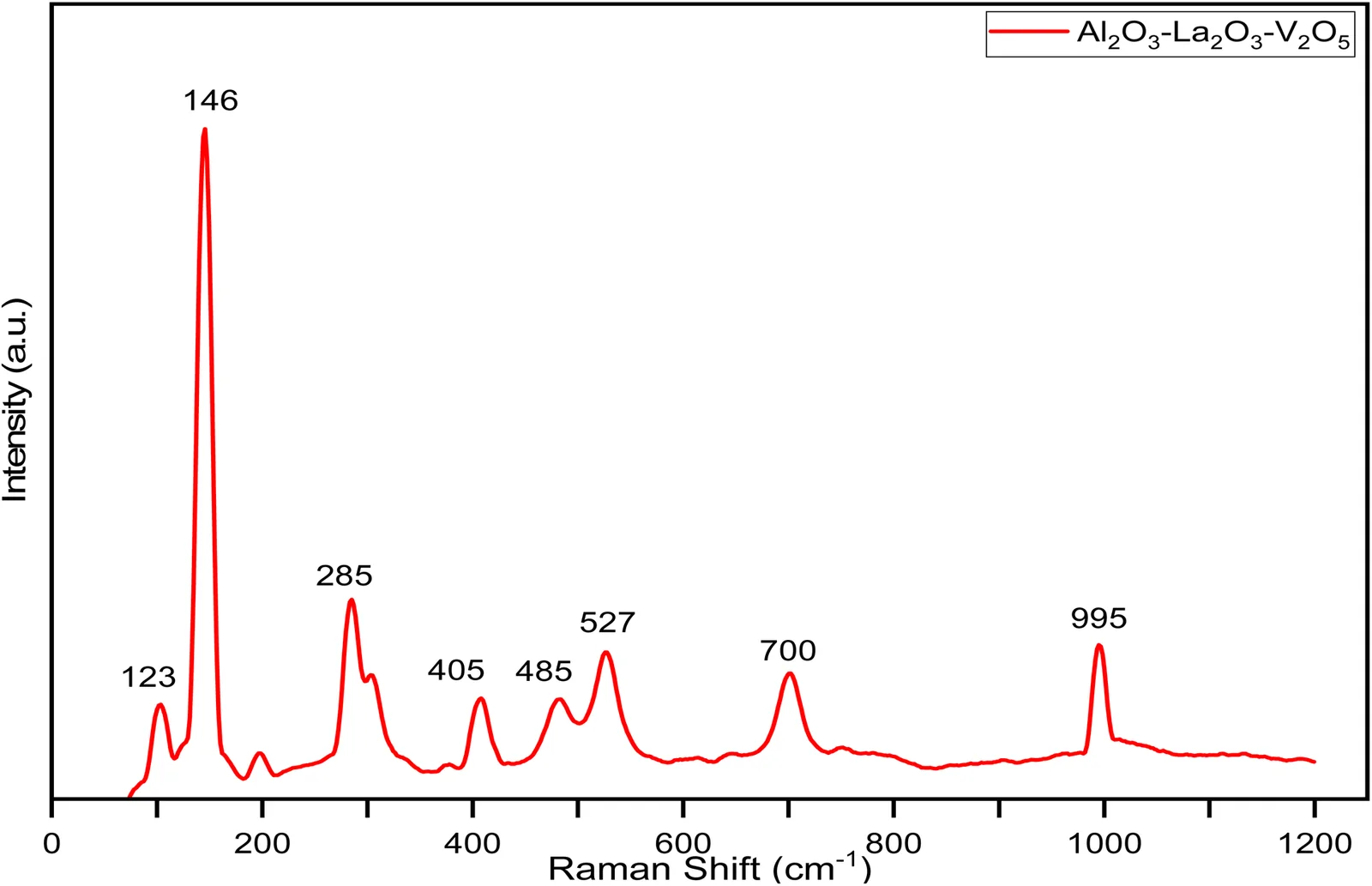
Raman spectroscopy of Al_2_O_3_–La_2_O_3_–V_2_O_5_ nanocomposites.

Raman spectroscopy was used to study the vibrational properties of the Al_2_O_3_–La_2_O_3_–V_2_O_5_ nanocomposites. The spectra (Fig. 9) showed unique peaks at 123, 146, 285, 405, 485, 527, 700, and 995 cm^−1^, each representing a specific vibrational mode of the constituent oxides. Low-frequency bands at 405 cm^−1^ correspond to lattice vibrations and La–O bonding modes.^66^ Peaks at 123, 146 and 285 cm^−1^ correspond to V–O–V bending and O–V–O deformation modes, indicating polyvanadate structures.

The peak at 485 cm^−1^ indicates symmetric V–O–V stretching, the 700 cm^−1^ peak represents the symmetric stretching of VO_4_ tetrahedra, suggesting the presence of orthovanadate phases like LaVO_4_. The signal at 995 cm^−1^ indicates terminal V=O stretching (vanadyl groups) and confirms the presence of V^5+^ species in the structure,^67^ most likely due to V_2_O_5_. Whereas the 527 cm^−1^ band indicates the Al–O vibrations.^68^ These assignments represent the production of mixed oxide phases and interactions involving vanadium, lanthanum, and aluminum oxides.

### 3.8 TEM analysis of Al_2_O_3_–La_2_O_3_–V_2_O_5_ nanocomposites

**Fig. 10:**
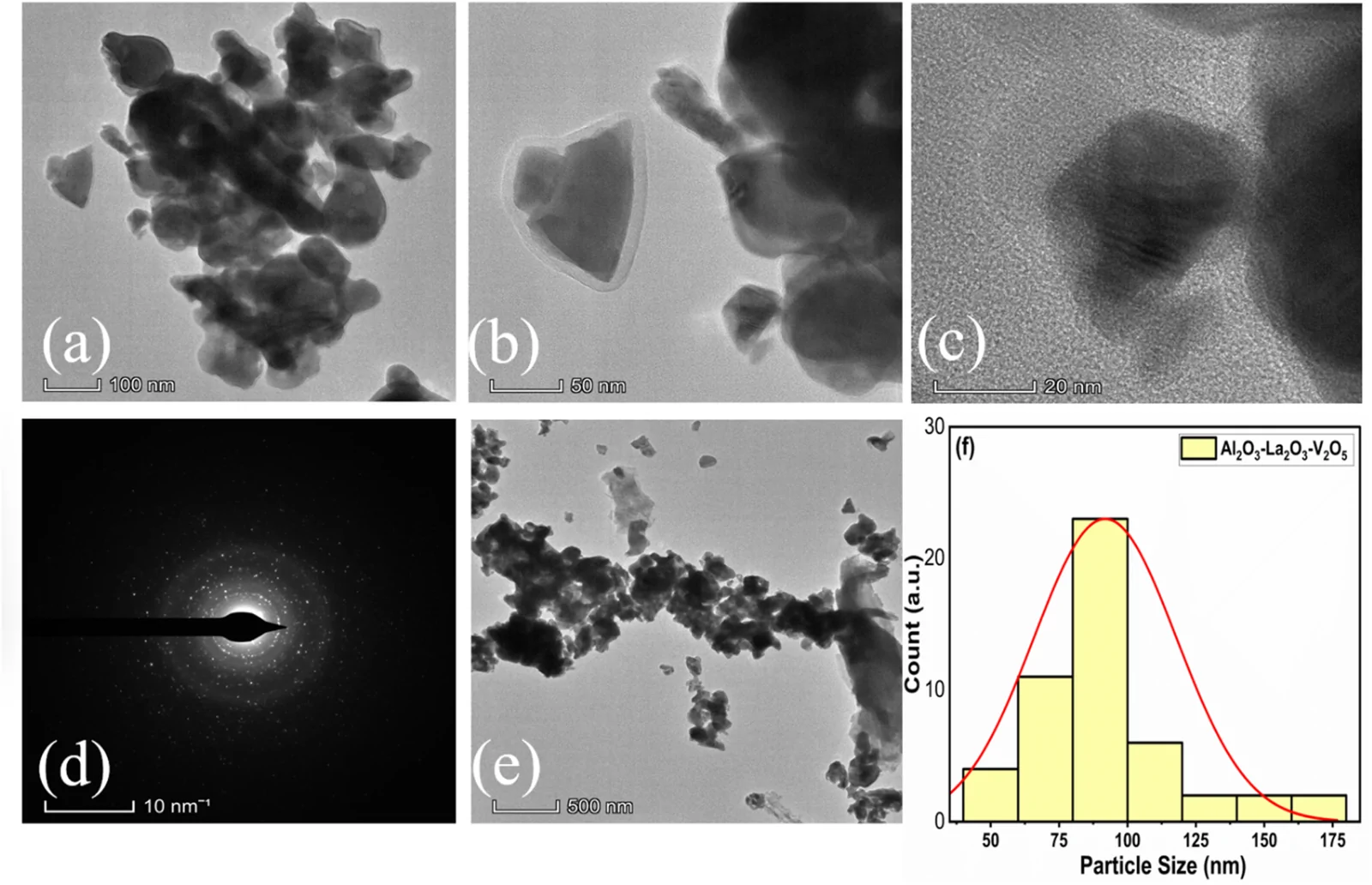
(a–e) TEM images of the as-prepared Al_2_O_3_–La_2_O_3_–V_2_O_5_ nanocomposites at different magnifications and (f) particle size distribution curve.

The TEM investigation confirmed the particle size, growth pattern, and crystallite distribution.^69^Fig. 10 depicts a TEM picture of synthesised catalyst. The TEM picture shows near sphere-shaped Al_2_O_3_–La_2_O_3_–V_2_O_5_ nano-catalyst generated during wet impregnation. The average diameter of the nanocomposite was around 91 nm which was calculated from the distribution curve.

The particles are also easily visible in TEM images, where they are assembled in pre-designed nanostructures *via* aggregation.^70^ The nanocrystallites aggregate into bigger particles. The TEM pictures of the Al_2_O_3_–La_2_O_3_–V_2_O_5_ nanocomposite at various magnifications are shown in Fig. 10(a and b). As is typical for metal oxide nanocomposites made using the wet-impregnation technique, the nanoparticles have an uneven shape and are grouped into clusters. The crystalline nature of the produced nanocomposite is confirmed by the HRTEM picture (Fig. 10(c)), which shows distinct lattice fringes. The sample's polycrystalline character is indicated by the concentric diffraction rings seen in the corresponding SAED pattern (Fig. 10(d)). While the particle size distribution histogram (Fig. 10(f)) shows that the particles are primarily located within the nanoscale range with a rather narrow size distribution, Fig. 10(e) further highlights the general morphology and distribution of the nanoparticles.

The sizes obtained from the multiple procedures are mutually consistent once it is realized that each explores a distinct structural aspect. XRD reflects the size of coherently diffracting crystalline domains and gave 43.2 nm (Scherrer), 49 nm (Monshi–Scherrer) and 58.2 nm (Williamson–Hall); the Williamson–Hall result is significantly bigger because it isolates the lattice-strain contribution (*ε* = 5.0 × 10^−4^) from size broadening. These crystallite dimensions coincide well with the principal particle size of ≈49 nm observed by SEM, showing that the particles visible by SEM are essentially single crystallites or small aggregates of a few crystallites. The TEM value, which is slightly higher at approximately 91 nm, is indicative of particles and small agglomerates. The apparent size of these entities is contingent upon the degree of aggregation and the imaging region. The slight disparity between the SEM and TEM readings consequently reflects the differing measurement principles and the identification of particle boundaries in an agglomerated sample rather than an actual structural mismatch.

### 3.9 BET analysis

**Fig. 11:**
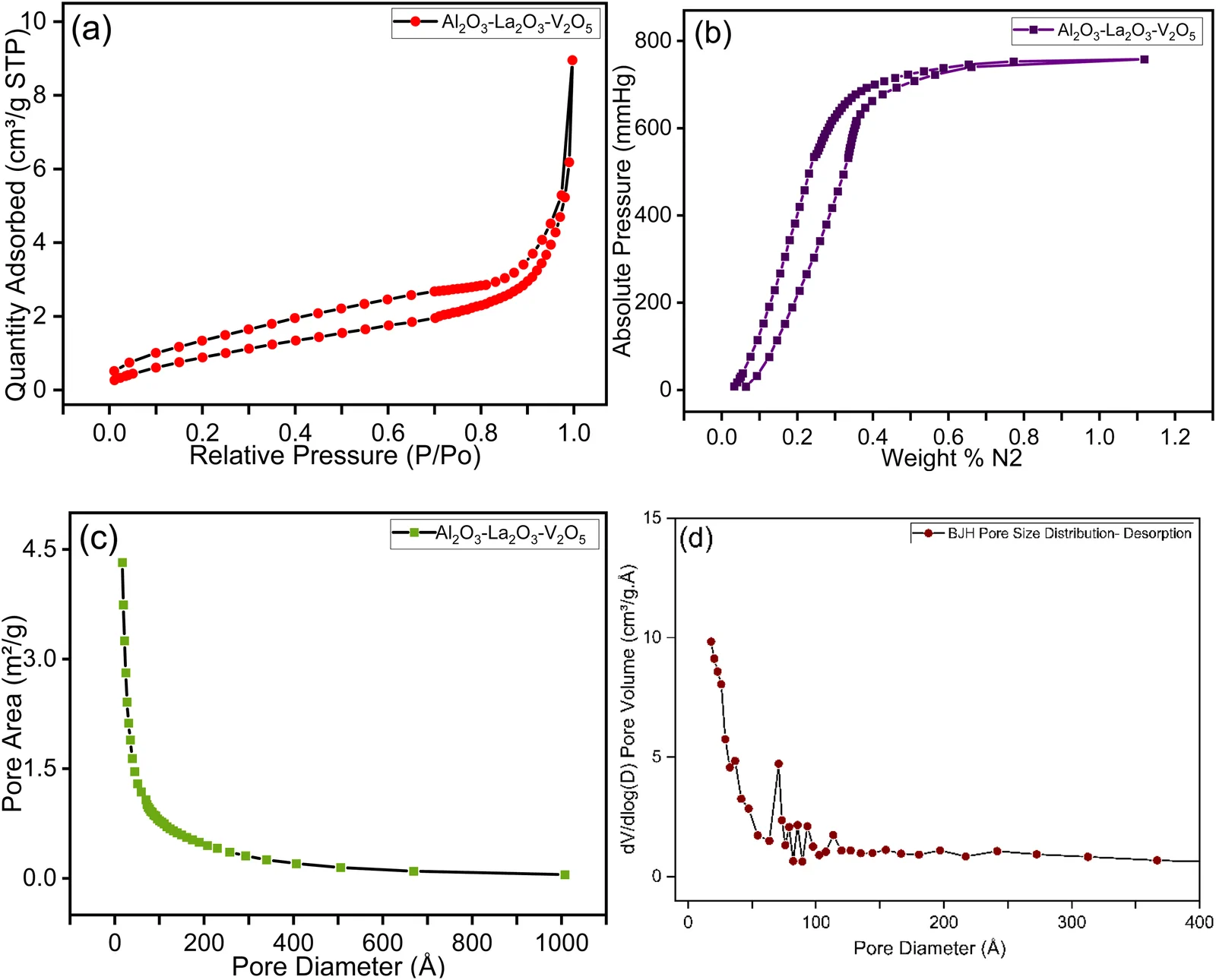
(a–d) Nitrogen adsorption–desorption isotherm and pore analyses of Al_2_O_3_–La_2_O_3_–V_2_O_5_ nanocomposite; (d) BJH pore size distribution.

The N_2_ adsorption–desorption isotherms and pore size distribution plots for Al_2_O_3_–La_2_O_3_–V_2_O_5_ nanocomposite under various circumstances are shown in Fig. 11. In Al_2_O_3_–La_2_O_3_–V_2_O_5_, which has a specific surface area of 3.8662 m^2^ g^−1^, a pore volume of 0.017 cm^3^ g^−1^, and a pore size of 89.944 Å. The mesoporous structure of the nanocomposite offers accessible active sites, which may improve its photocatalytic performance, even though the surface area is relatively small. The BJH pore size distribution is shown in Fig. 11(d), showing a narrow range with an average pore size of 58.675 Å for Al_2_O_3_–La_2_O_3_–V_2_O_5_ nanocomposite, which corresponds to the mesoporous structure categorized as type IV isotherm with an IUPAC hysteresis loop.^71^

### 3.10 Photocatalytic activity of Al_2_O_3_–La_2_O_3_–V_2_O_5_ nanocomposites

#### 3.10.1 Effect of concentration

**Fig. 12:**
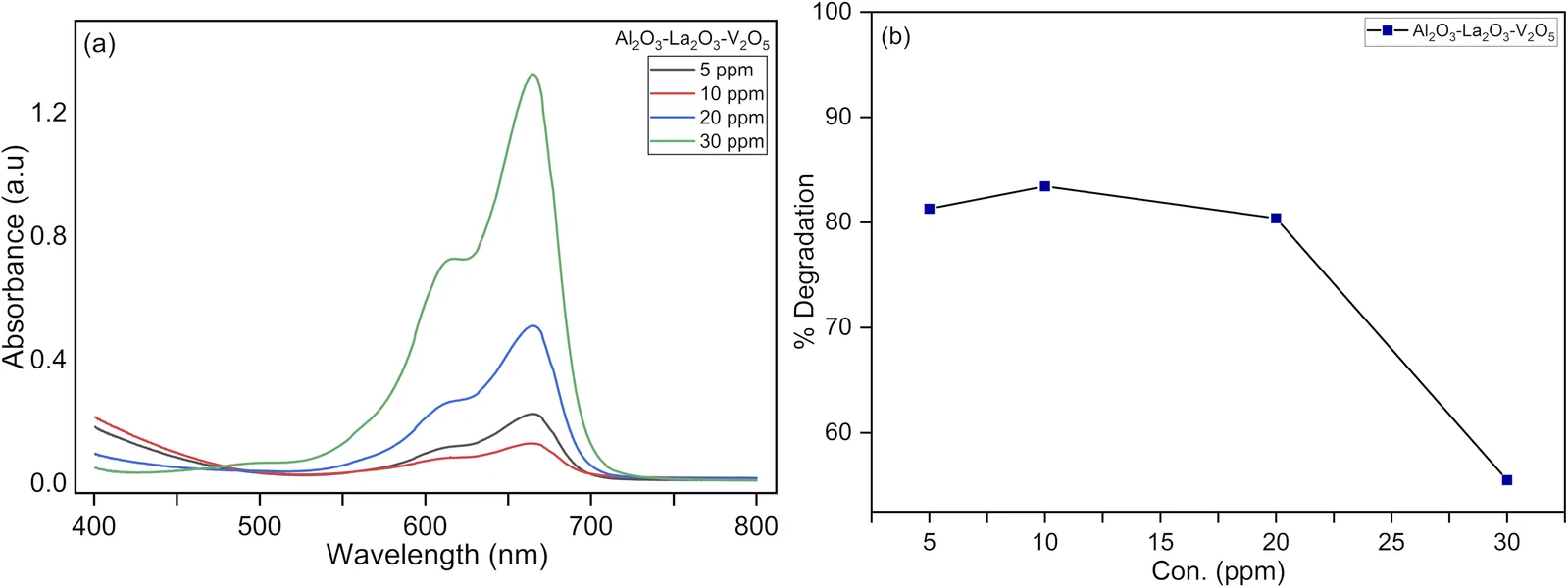
(a) The photocatalytic activity based on concentration and (b) degradation percentage of Al_2_O_3_–La_2_O_3_–V_2_O_5_ nanocomposite.

Four distinct concentrations of photocatalysts were created by varying the catalyst concentration from 5 to 30 ppm. Interestingly, 10 ppm photocatalyst solution outperformed other solutions during MB decolorization under sunlight. At a dye concentration of 10 ppm, the nanocomposite achieved approximately 83% MB degradation within 2 hours, following pseudo-first-order kinetics and outperforming earlier La_2_O_3_/rGO systems (73.7%).^72^ The 10 ppm concentration yielded the highest degradation (83%) after 2 hours of sunlight irradiation. At 2 hours of irradiation, over 83% of the MB was decolorized which is shown below through Fig. 12.

The maximum MB degradation at 10 ppm is attributed to excellent light penetration and effective interaction between dye molecules and photo-generated reactive species; greater concentrations suffer from light shielding, and lower concentrations contain insufficient dye molecules.^73^ As the concentration rises to 20 ppm, the efficiency falls slightly to 80%, indicating that the active sites on Al_2_O_3_–La_2_O_3_–V_2_O_5_ nanocomposite begin to compete with the increased pollutant burden. At 30 ppm, the efficiency further declines to 56%, suggesting a more marked limitation in degradation capacity, possibly due to active site saturation, light penetration, and competing intermediates.^74^

#### 3.10.2 Effect of pH

**Fig. 13:**
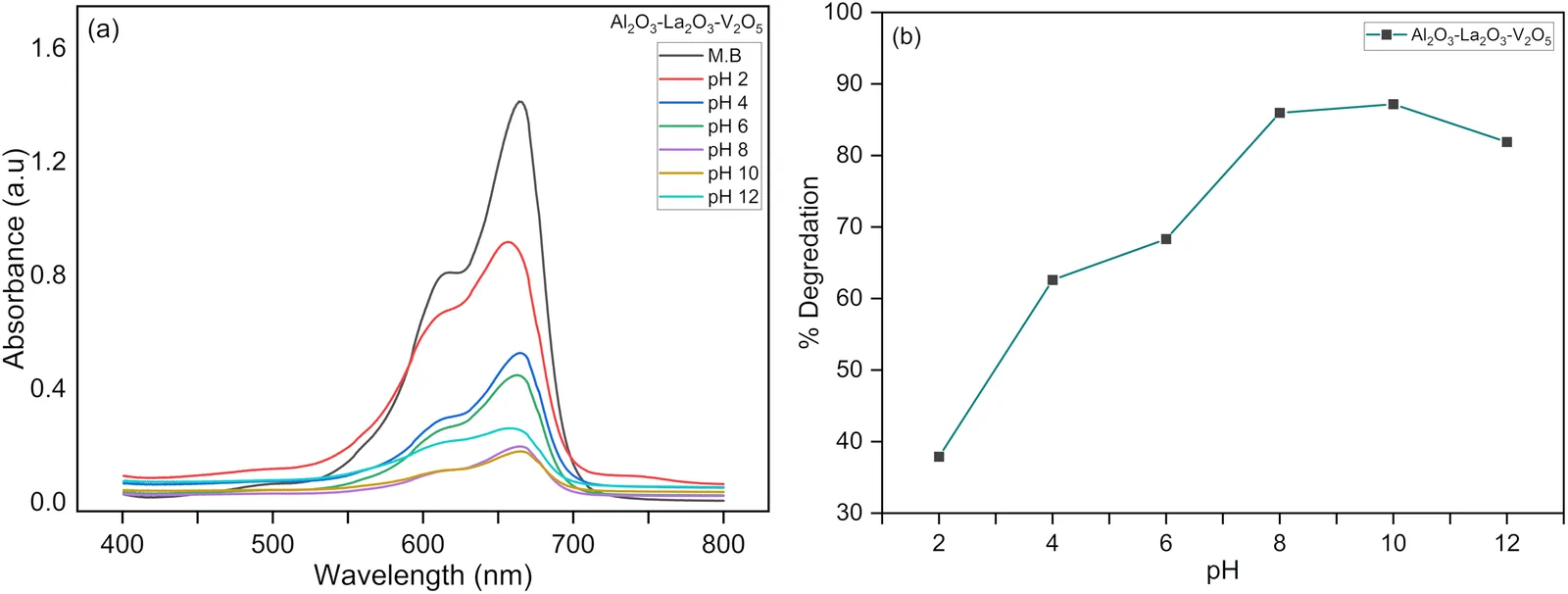
(a) The photocatalytic activity based on pH and (b) degradation percentage of Al_2_O_3_–La_2_O_3_–V_2_O_5_ nanocomposite.

As illustrated in Fig. 13, the photocatalytic degradation of MB was nearly complete within 120 min of reaction time. The degradation efficiency of MB under photocatalytic treatment shows a pH-dependent pattern. The maximum clearance rate was found at pH 10 (88.5%). Fig. 13 shows that the pH range (8.0–10.0) is optimal for catalyst–dye interactions and reactive species formation, resulting in approximately 88% elimination. Operating in this alkaline range enhances catalyst dye interaction and reactive-species formation during treatment.^75^

The photocatalytic activity of the nanocomposite was strongly pH dependent, with 88.5% degradation at pH 10. The current ternary architecture outperforms g-C_3_N_4_/V_2_O_5_/PANI composites, which have a maximum efficiency of 70% under similar alkaline circumstances,^76^ indicating higher catalytic efficacy.

#### 3.10.3 Effect of time

The photocatalytic capabilities of the produced Al_2_O_3_–La_2_O_3_–V_2_O_5_ nanocomposite were studied by degrading MB. UV-vis transmission density bands were analyzed to show the effect of irradiation duration on the degradation of MB dye. The degradation percentage was found by measuring the absorbance.

**Fig. 14:**
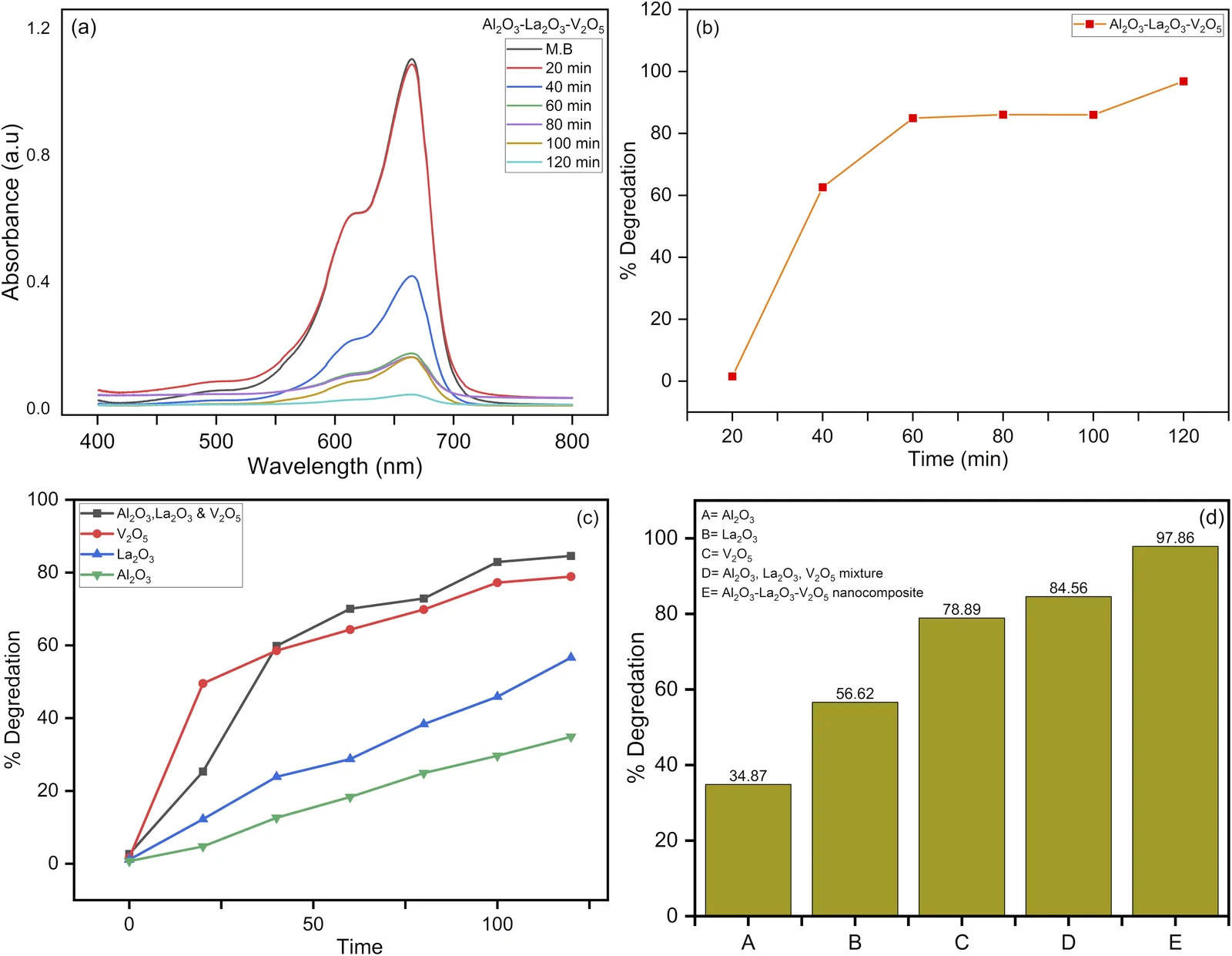
(a) The photocatalytic activity and (b) degradation of Al_2_O_3_–La_2_O_3_–V_2_O_5_ nanocomposite and (c) Al_2_O_3_, La_2_O_3_, V_2_O_5_ and their physical mixture based on time and (d) their bar comparison.

Under sunlight, the photocatalytic performance of the produced samples was investigated by measuring the decrease in the intensity of the maximum absorption peaks at 20-minute intervals from 0 to 120 minutes. Fig. 14 shows the absorption spectra and degradation rate of MB. The nanocomposite exhibited a degradation efficiency of 2.28% at 20 min, which increased progressively to 97.86% at 120 min. Under visible light, the nanocomposite demonstrated a degradation efficiency of 97.86% toward methylene blue, this performance surpasses several reported photocatalysts under comparable conditions: Al_2_O_3_/Fe_2_O_3_ (75.10%),^77^ Fe–AC/TiO_2_ (85%),^78^ ZnO/WO_3_ (93.8%),^79^ Zn_3_(VO_4_)_2_ (87%),^80^ NiO/Ag/TiO_2_ (93.15%).^81^ The increased activity emphasizes the electrical and interfacial synergy of the ternary system (Table 2).

**Table 2:** Comparison of the photocatalytic degradation performance of the Al_2_O_3_–La_2_O_3_–V_2_O_5_ nanocomposite with reported photocatalysts for methylene blue degradation

No.	Photocatalyst	Degradation (%)
1	Al_2_O_3_/Fe_2_O_3_	75.10
2	Fe–AC/TiO_2_	85
3	ZnO/WO_3_	93.80
4	Zn_3_(VO_4_)_2_	87
5	NiO/Ag/TiO_2_	93.15
Photocatalyst	Al_2_O_3_–La_2_O_3_–V_2_O_5_	97.86

To improve photocatalytic performance, adjusting the band gap by using controlled doping or forming heterostructures can shift the absorption edge to longer wavelengths and increase visible-light use.^82,83^ Doping increases exterior charge and surface area while shifting the band gap to red, hence improving photocatalytic performance.^84,85^ As a result, the MB solution turned completely colorless within 120 minutes of sunlight exposure while using the nanocomposite.

A physical mixture of Al_2_O_3_, La_2_O_3_, V_2_O_5_ was created. The physical combination only destroyed 84.56% of MB where Al_2_O_3_ (34.87%), La_2_O_3_ (56.62%) and V_2_O_5_ (78.89) under the same reaction conditions while the Al_2_O_3_–La_2_O_3_–V_2_O_5_ nanocomposite achieved 97.86% degradation. The development of intimate heterointerfaces during the wet-impregnation process, which promote effective charge separation and interfacial electron transmission while inhibiting electron–hole recombination, is responsible for the nanocomposite's outstanding performance. As a result, more reactive oxygen species are produced, which improves MB's photocatalytic degradation. This observation demonstrates that the enhanced activity is not the consequence of the constituent oxides' simple physical mixing but rather of their synergistic interaction.^86^

##### 3.10.3.1 Mechanistic investigation by radical trapping experiments

**Fig. 15:**
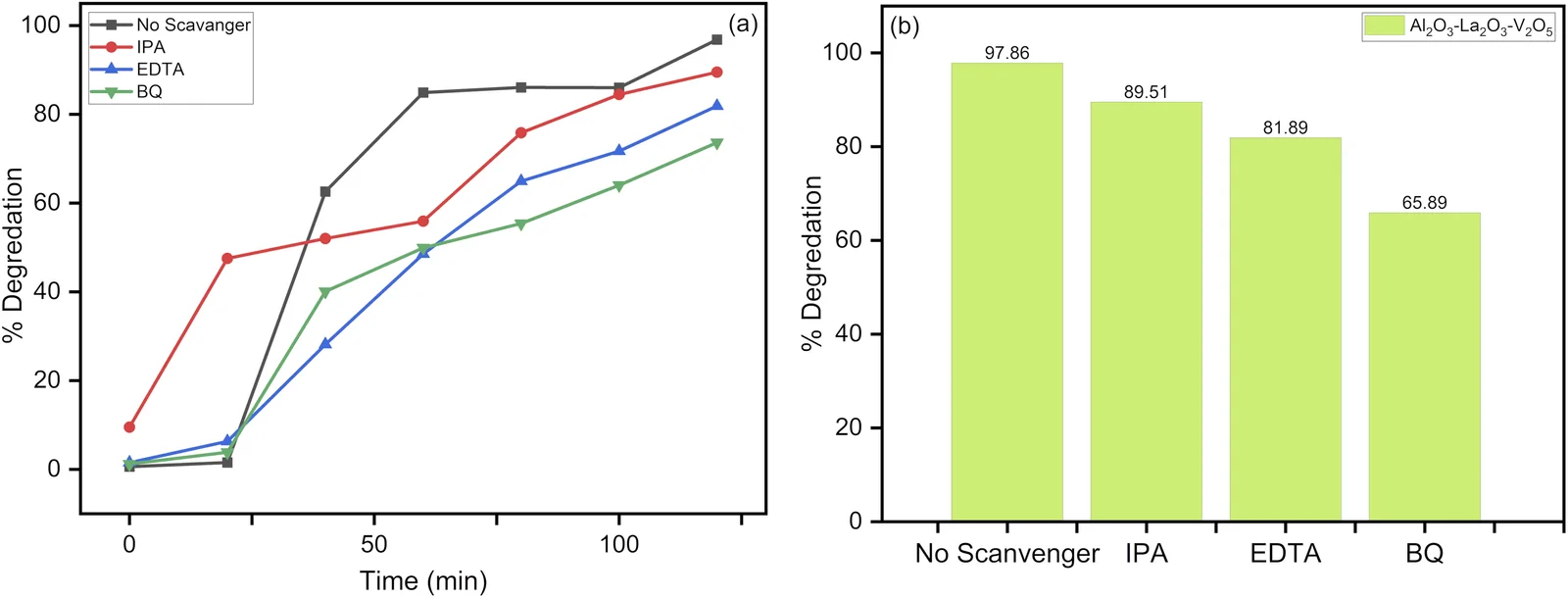
(a) Radical trapping experiments for MB photodegradation and (b) degradation bar diagram.

To determine the main reactive species in charge of the photocatalytic degradation of methylene blue, radical trapping studies were conducted. Na-EDTA, *p*-benzoquinone (BQ), and isopropyl alcohol (IPA) were used as scavengers for photogenerated holes (h^+^), ˙O_2_^−^ radicals, and ˙OH radicals,^87^ respectively. ˙O_2_^−^ radicals are the main reactive species involved in the photocatalytic process, as seen by Fig. 15, where the addition of BQ caused the most noticeable drop in the degradation efficiency when compared to IPA and Na-EDTA. ˙OH radicals and h^+^ may also contribute to the degradation, albeit to a lesser degree, based on the comparatively smaller inhibition seen in the presence of IPA and Na-EDTA. These results show that the superoxide radical-mediated oxidation pathway is predominantly responsible for the photocatalytic degradation of methylene blue over the Al_2_O_3_–La_2_O_3_–V_2_O_5_ nanocomposite.

#### 3.10.4 Effect of weight

**Fig. 16:**
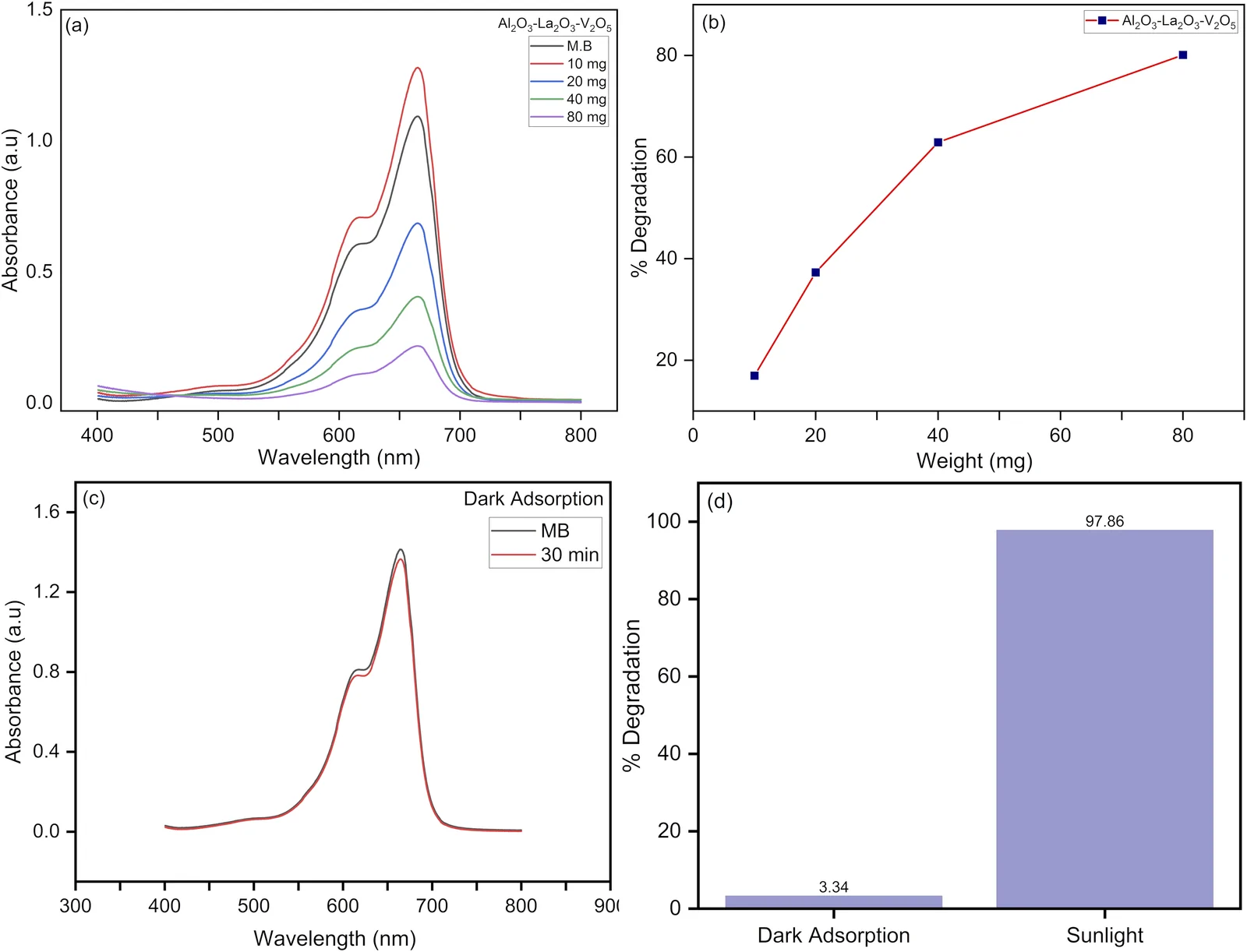
(a) Photocatalytic performance of Al_2_O_3_–La_2_O_3_–V_2_O_5_ nanocomposite: effect of catalyst weight, (b) degradation efficiency, and (c) dark-adsorption profile of methylene blue and (d) comparison of dark-adsorption and sunlight-driven degradation activity.

The degradation of MB in sunlight was used to evaluate the photocatalytic activity of the Al_2_O_3_–La_2_O_3_–V_2_O_5_ nanocomposite. The absorption spectra of the MB aqueous solution containing 10 mg, 20 mg, 40 mg, and 80 mg of Al_2_O_3_–La_2_O_3_–V_2_O_5_ nanocomposite–photocatalysts are displayed in Fig. 16. Under sunlight, the concentration of MB dropped by just 4.14% without photocatalysts. Because of the nanometric dimensions, the concentration of MB dropped during the irradiation period when Al_2_O_3_–La_2_O_3_–V_2_O_5_ nanocomposite was used as a photocatalyst. The MB degrading capability of the nanocomposite photocatalysts was higher demonstrating that the inclusion of Al_2_O_3_–La_2_O_3_–V_2_O_5_ nanocomposite improved the photocatalytic performance.

The predicted photocatalytic degradation efficiencies were 18.45%, 38.85%, 68.80% and 80.02% based on the experimental results. The steeper slope of the curves in Fig. 16 indicates that the breakdown rate of MB was higher in the case of the nanocomposites with higher amount. Because more active sites were available and more reactive oxygen species were produced, the photocatalytic degradation efficiency rose as the Al_2_O_3_–La_2_O_3_–V_2_O_5_ nanocomposite loading increased. Within the examined catalyst dose range, no light-shielding effect was seen. The nanocomposite demonstrated 80.02% methylene blue degradation, outperforming Ca–TiO_2_ (79.65%) and pure TiO_2_ (46.15%) under the same conditions,^88^ indicating improved photocatalytic activity.

Al_2_O_3_–La_2_O_3_–V_2_O_5_ nanocomposite can accelerate dye degradation and increase the rate of electron transport.^89^ Charge carriers that have evaded annihilation move to the nanocomposite surface during photocatalysis and start reactions with the species that are adsorbed on the surface. While the electrons react with dissolved oxygen to produce superoxide or hydroperoxide radicals, the holes react with H_2_O molecules to produce hydroxyl radicals. Each of this species aids in the methylene blue dye's breakdown.^90^

To achieve adsorption–desorption equilibrium, the catalyst suspension was agitated in the dark for half an hour prior to photocatalytic irradiation. The Al_2_O_3_–La_2_O_3_–V_2_O_5_ nanocomposite showed just 3.34% methylene blue elimination during the dark period, suggesting minimal dye adsorption onto the catalyst surface. The degradation efficiency significantly increased to 97.86% after further solar light irradiation, indicating that photocatalytic degradation rather than dark adsorption was primarily responsible for the elimination of methylene blue.

#### 3.10.5 Kinetic study of Al_2_O_3_–La_2_O_3_–V_2_O_5_

By measuring the absorbance of the methylene blue solution, one may determine the concentration of the solution depending on the degradation efficiency of the catalyst. Eqn (10)–(12) fitting reveals that the MB degradation process follows first-order reaction kinetics:10
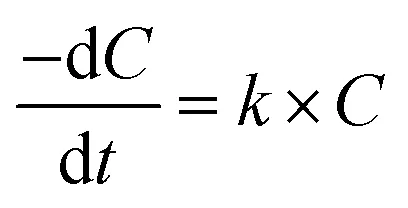
11
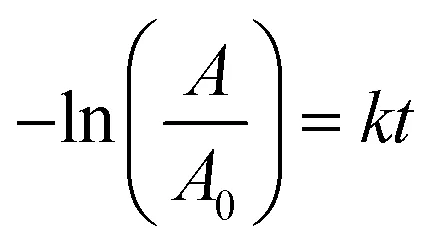
12
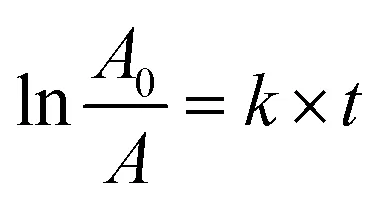
where *A*, *A*_0_, *k*, and *t* stand for the MB concentration of “*t*” time, the dye's starting concentration, the reaction rate constant (min^−1^), and the duration of photocatalytic degradation,^91^ respectively.

**Fig. 17:**
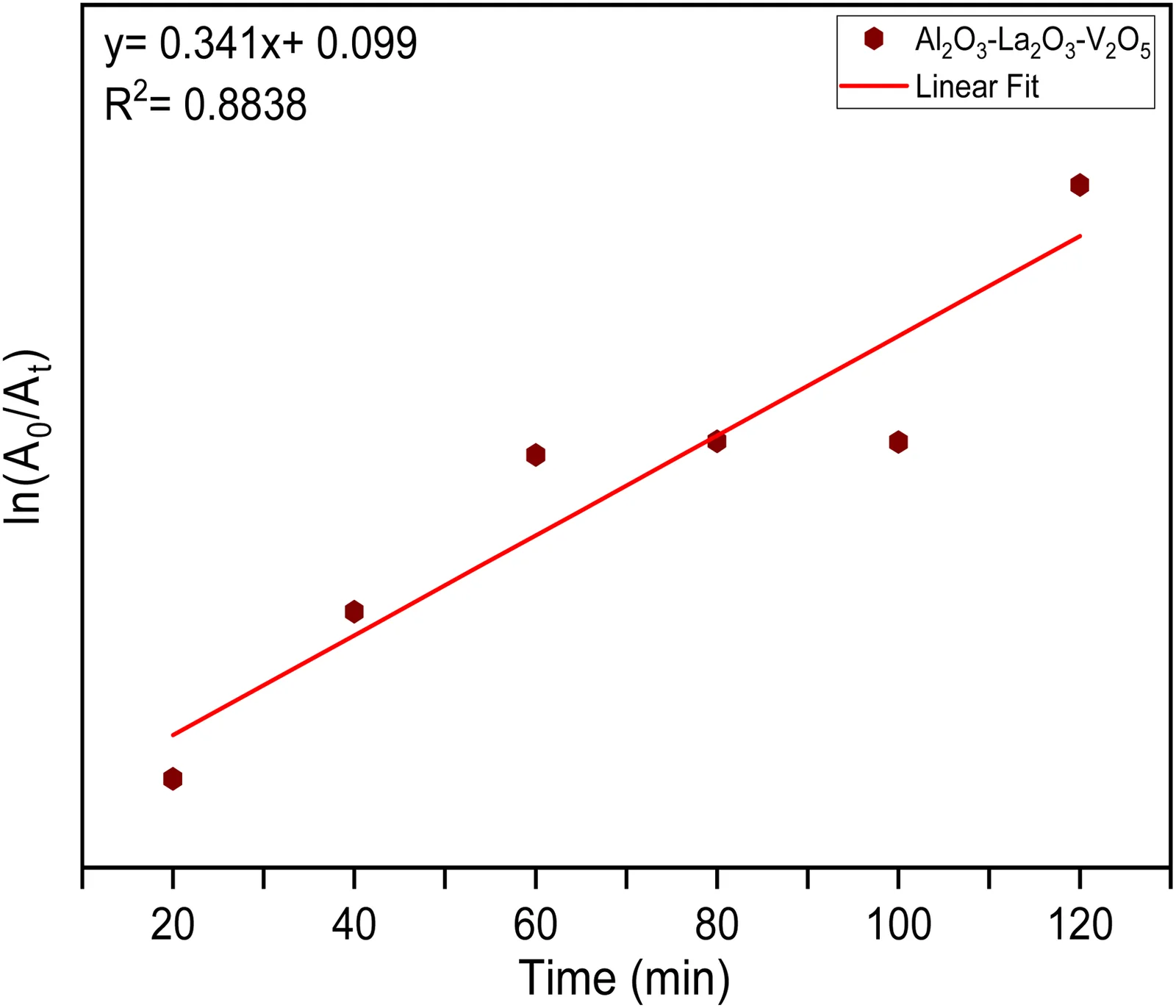
Kinetic study of Al_2_O_3_–La_2_O_3_–V_2_O_5_ nanocomposite.

Fig. 17 displays the results of fitting the reaction kinetics of each set of photocatalytic degradation tests. For this experiment, a fitting analysis was carried out up to a 90% degree of deterioration. Fig. 17 displays the fitting data for the experimental findings. Since *R*^2^ is more than 0.88 in every result, the MB degradation experiment's ln(*A*_0_/*A*) exhibits a linear relationship with time *t*, which is a first-order reaction and satisfies the equation above. It is evident that the sample that degrades MB solution the best by photocatalysis.

#### 3.10.6 Point of zero charge study of Al_2_O_3_–La_2_O_3_–V_2_O_5_ nanocomposites

The point of zero charge (PZC) is a fundamental description of an oxide surface. The pH at PZC of the prepared samples was determined using the batch equilibrium technique (solid addition method^92^), as shown in the protocol below. Several samples of 10 mg Al_2_O_3_–La_2_O_3_–V_2_O_5_ nanocomposites were injected into 30 mL of 0.1 M KCl solution at various initial pH levels (2, 4, 6, 7, 8, 10, and 12). The initial pH (pH_i_) of all suspensions was adjusted using 0.1 M KOH and/or 0.1 M HCl solutions. The suspensions with known pH_i_ were then agitated at room temperature for 8 hours to equilibrate them. Following that, the samples were filtered, and the final pH values (pH_f_) were determined after 8 hours. In the photocatalytic system based on Al_2_O_3_–La_2_O_3_–V_2_O_5_ nanocomposite, when the net total particle charge at a specific pH value is zero, the isoelectric point, also known as the point of zero charge (PZC), is attained. During photocatalysis, the PZC of the nanocomposite is an important parameter used to indicate the photocatalyst's changing charge surface. This study determined the PZC of the Al_2_O_3_–La_2_O_3_–V_2_O_5_ nanocomposite in the pH range of 2–12. When the pH of a solution surpasses the PZC value, the compound enables the adsorption of positively charged particles. The nanocomposite makes it easier for negatively charged dyes (contaminants) to adsorb when the pH is lower than the PZC.^93^ Controlling the solution pH changes the surface charge of the photocatalyst. This allows for the selective breakdown of certain dyes. In our case, MB is a well-known cationic dye, and a pH value larger than the PZC value is more advantageous for its degradation.

**Fig. 18:**
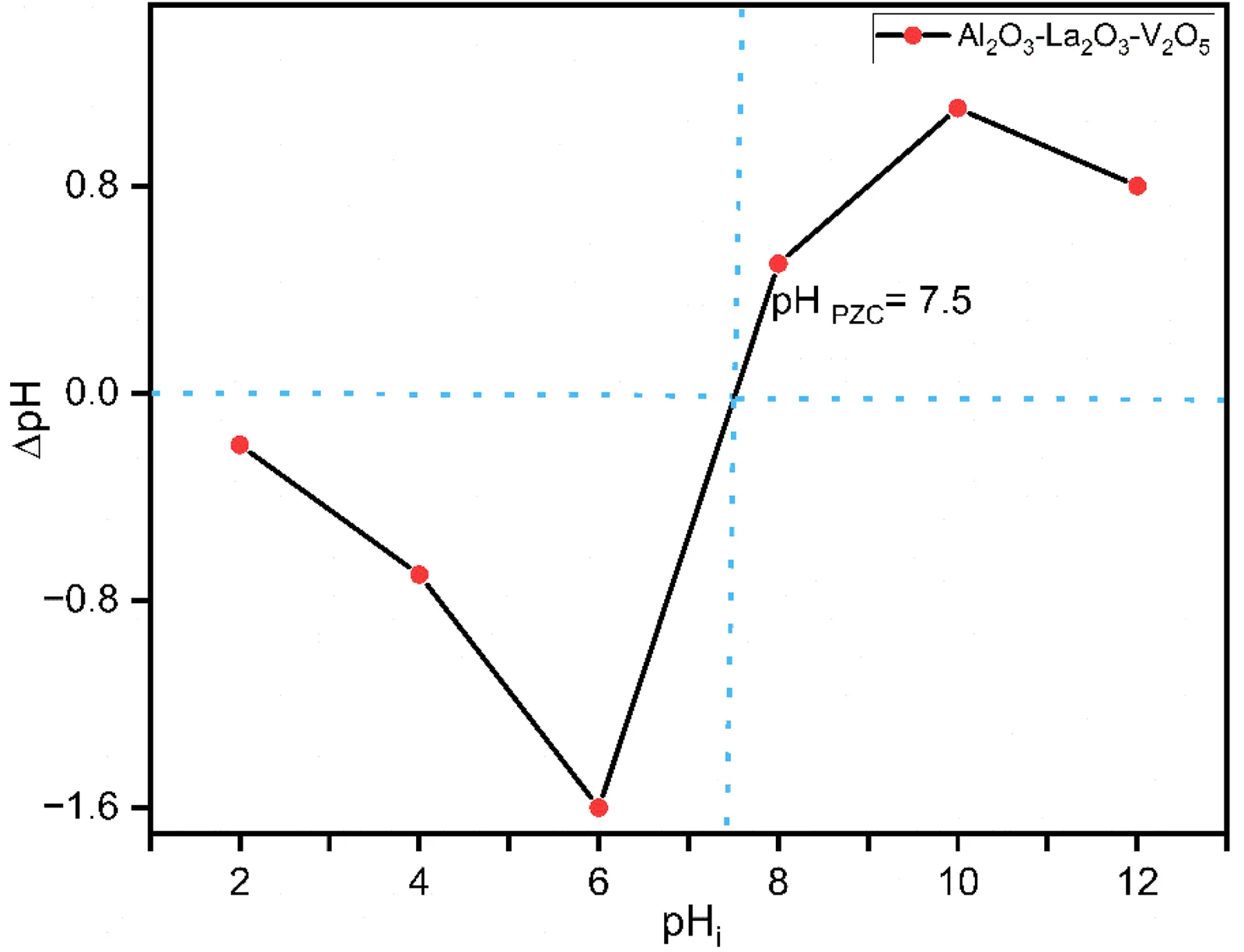
Point of zero charge study of Al_2_O_3_–La_2_O_3_–V_2_O_5_ nanocomposite.

The difference between the initial and final pH_s_ (ΔpH = pH_f_ − pH_i_) was computed and plotted against pH_i_. The pH PZC value of Al_2_O_3_–La_2_O_3_–V_2_O_5_ nanocomposites is represented by the place where the curve intersects at ΔpH = zero. Fig. 18 indicated that the PZC of Al_2_O_3_–La_2_O_3_–V_2_O_5_ was estimated to be approximately 7.5.

## 4 Conclusion

The synthesis and systematic characterization of a novel ternary Al_2_O_3_–La_2_O_3_–V_2_O_5_ nanocomposites was accomplished. The creation of nanoscale, nearly spherical particles with high crystallinity was confirmed by structural and morphological investigations. XRD fitting using the Scherrer, Monshi–Scherrer, and Williamson–Hall models revealed an average crystallite sizes of 43.2 nm, 49 nm and 58.2 nm respectively. While FTIR and Raman spectroscopy revealed the presence of distinctive functional groups, SEM/EDS and TEM studies confirmed the expected elemental composition and nanoscale shape. The catalyst exhibited direct and indirect optical band gaps of 2.72 eV and 1.77 eV, respectively, confirming visible-light photoactivity. Using methylene blue as a probe molecule, photocatalytic evaluation showed excellent activity, attaining 97.86% degradation in 120 minutes when exposed to natural sunshine and following pseudo-first-order reaction kinetics. Thermogravimetric analysis verified strong thermal stability, suggesting resilience at high temperatures, whereas BET analysis showed moderate surface area and porosity.

Overall, the findings show that Al_2_O_3_, La_2_O_3_, and V_2_O_5_ work in concert to produce a robust and effective photocatalyst with significant potential for sunlight-driven catalytic applications, especially in photocatalytic degradation of methylene blue and related organic dye pollutants.

## Author contributions

Abdullah Al Mamun: investigation, methodology, writing – original draft. Swapan Kumer Ray: investigation, formal analysis, methodology, conceptualization, visualization. Md. Omar Sha Rafi: conceptualization, methodology, writing – review and editing, supervision. Shabiba Parvin Shandhi: data curation, investigation. Riyadh Hossen Bhuiyan: investigation, validation. Fariha Chowdhury: resources, investigation. Tanvir Muslim: conceptualization, project administration, funding acquisition, supervision.

## Conflicts of interest

There are no conflicts to declare.

## Data Availability

The data supporting the findings of this study are available within the article and from the corresponding author upon reasonable request.
